# BDNF/proBDNF Interplay in the Mediation of Neuronal Apoptotic Mechanisms in Neurodegenerative Diseases

**DOI:** 10.3390/ijms26104926

**Published:** 2025-05-21

**Authors:** Marina Mitrovic, Dragica Selakovic, Nemanja Jovicic, Biljana Ljujic, Gvozden Rosic

**Affiliations:** 1 Department of Medical Biochemistry, Faculty of Medical Sciences, University of Kragujevac, 34000 Kragujevac, Serbia; mitrovicmarina34@gmail.com; 2 Department of Physiology, Faculty of Medical Sciences, University of Kragujevac, 34000 Kragujevac, Serbia; grosic@fmn.kg.ac.rs; 3 Department of Histology and Embryology, Faculty of Medical Sciences, University of Kragujevac, 34000 Kragujevac, Serbia; nemanjajovicic.kg@gmail.com; 4 Department of Genetics, Faculty of Medical Sciences, University of Kragujevac, 34000 Kragujevac, Serbia; bljujic74@gmail.com

**Keywords:** neurotrophins, neurotrophin receptors, apoptosis, Alzheimer’s disease, Parkinson’s disease, Huntington’s disease

## Abstract

The neurotrophic system includes neurotrophins, such as brain-derived neurotrophic factor (BDNF) and its precursor proBDNF, which play conflicting roles in neuronal survival and apoptosis, with their balance having a significant impact on neurodegenerative outcomes. While BDNF is widely acknowledged as a potent neurotrophin that promotes neuronal survival and differentiation, its precursor, proBDNF, has the opposite effect, promoting apoptosis and neuronal death. This review highlights the new and unique aspects of BDNF/proBDNF interaction in the modulation of neuronal apoptotic pathways in neurodegenerative disorders. It systematically discusses the cross-talk in apoptotic signaling at the molecular level, whereby BDNF activates survival pathways such as PI3K/Akt and MAPK/ERK, whereas proBDNF activates p75NTR and sortilin to induce neuronal apoptosis via JNK, RhoA, NFkB, and Rac-GTPase pathways such as caspase activation and mitochondrial injury. Moreover, this review emphasizes the factors that affect the balance between proBDNF and BDNF levels within the context of neurodegeneration, including proteolytic processing, the expression of TrkB and p75NTR receptors, and extrinsic gene transcription regulators. Cellular injury, stress, or signaling pathway alterations can disrupt the balance of BDNF/proBDNF, which may be involved in apoptotic-related neurodegenerative diseases like Alzheimer’s, Parkinson’s, and Huntington’s diseases. This review provides a comprehensive framework for targeting neurotrophin signaling in the development of innovative therapies for neuronal survival and managing apoptotic-related neurodegenerative disorders, addressing the mechanistic complexity and clinical feasibility of BDNF/proBDNF interaction.

## 1. Introduction

BDNF and proBDNF play a critical role in regulating neuronal apoptotic mechanisms in neurodegenerative disorders, including Alzheimer’s (AD), Parkinson’s (PD), and Huntington’s diseases (HD). Despite the extensive literature detailing the individual roles of BDNF and proBDNF in neuronal health, there is a lack of integrated analysis that connects their interplay in regulating neuronal apoptosis across multiple levels in distinct neurodegenerative disorders. This review aims to systematically summarize current knowledge from the existing literature regarding the roles of BDNF and proBDNF in neuronal apoptosis. It will also provide a comprehensive and mechanistically detailed analysis of how the interplay between BDNF and proBDNF connects to their regulatory mechanisms across gene expression, receptor signaling, and proteolytic processing, leading to apoptotic outcomes in the specific contexts of neurodegenerative diseases such as AD, PD, and HD. Notably, we evaluate the BDNF/proBDNF ratio not only as a mechanistic switch between survival and cell death, but also as a potential diagnostic and predictive biomarker for neurodegenerative disease progression. Additionally, we assess the therapeutic potential and practical applicability of modifying this ratio in humans, focusing on significant challenges like delivery barriers, receptor specificity, and protease targeting. This review synthesizes molecular knowledge with clinical relevance, suggesting a new conceptual framework that may influence the advancement of future-generation neuroprotective strategies and therapeutic breakthroughs.

### 1.1. The Neurotrophic System

Neurotrophic factors (NTFs) and their receptors are a part of the complex neurotrophic system, which forms the basis for neuronal development, survival, and plasticity in both the CNS and PNS. In this sense, NTFs are considered natural proteins released by neurons, supporting cells responsible for maintaining, regenerating, and growing neurons. They also play a role in enhancing the expression of neural repair genes during neurodegeneration [1,2]. Some neurotrophins, like nerve growth factor (NGF) and brain-derived neurotrophic factor (BDNF), play a critical role in the regulation of apoptosis and the survival of neurons through complex signaling, thereby highlighting their dual role in neurobiology [3,4]. The normal physiological function of NTFs can be altered in the context of pathological diseases. In fact, NTF imbalances—either in transportation rate or baseline expression levels—can cause neuronal death and degeneration, which can lead to apoptotic-related neurological disorders like neuropsychiatric, neurodegenerative, and neurotraumatic conditions [5,6,7,8,9].

Researchers have identified approximately 50 distinct types of NTFs over the years. NTFs can be classified into four primary families based on modern biological and molecular research: neurotrophins, GDNF family ligands (GFLs), neuroprotective cytokines, and the CDNF/MANF family [10].

### 1.2. Neurotrophins

The neurotrophin (NT) family is the first neurotrophic family identified, and it includes nerve growth factors (NGFs), brain-derived neurotrophic factors (BDNFs), neurotrophin-3 (NT3), neurotrophin-4/5 (NT4/5), neurotrophin-6 (NT-6), and neurotrophin-7 (NT-7), among others [11]. Neurotrophins were originally described as survival factors for sympathetic and sensory neurons in the CNS and PNS. A mature neurotrophin is produced from a pro-neurotrophin, which comprises the mature protein and the prodomain. Multiple enzymes participate in the cleavage and processing of pro-neurotrophins, including furin in the Golgi apparatus, proconvertases in the dense core vesicle, and plasmin extracellularly [12,13]. Neurotrophins act mainly via two cell surface receptors: p75NTR and the TrkB receptors. By binding to neurotrophins, whether alone or in combination, these receptors can activate various signaling pathways, thereby producing complex physiological effects [4].

### 1.3. Neurotrophins and Trk Receptors

Neurotrophins bind mainly to Trk receptors, which consist of three transmembrane receptor tyrosine kinases: TrkA, TrkB, and TrkC (Table 1) [5,14]. These receptors have a modular structure with three primary domains: an extracellular domain for ligand binding and dimerization, a transmembrane domain for binding to the cell membrane, and an intracellular kinase domain for activating downstream signaling pathways [15]. Neurotrophins engage Trk receptors through their extracellular domain, resulting in dimerization and trans-autophosphorylation of specific tyrosine residues. These phosphorylated residues serve as docking sites for intracellular adaptor proteins, triggering downstream signaling cascades through phospholipase C-γ, PI3K, and MAPK pathways [16].

NGF, the first neurotrophin discovered [17], mainly interacts with TrkA to activate pathways that enhance the growth, differentiation, and survival of cholinergic neurons in the basal forebrain [18,19,20]. In contrast, BDNF and NT4/5 preferentially bind to TrkB in the central nervous system, facilitating growth, survival, differentiation, synaptic plasticity, and neuronal morphogenesis [21]. NT3 interacts with the TrkC (Ntrk3) receptor to regulate neuronal survival, assist differentiation, and promote new neuron and synapse growth [22,23]. Neurotrophins can cross-activate with Trk receptors with lower affinity to activate complex signaling cascades. Although specific pairings exist, neurotrophins can also interact with other Trk receptors with lower affinity. NT-3 may interact with Trk-A and Trk-B receptors in their alternate spliced forms, though with lower affinity than NGF and BDNF [24]. NT-4 may also interact with TrkA receptors [24]. Additionally, BDNF, NT-3, and NT-4 can interact with modified TrkBT1 receptors, which negatively impact full-length TrkB [25]. NT-6, expressed by various species, interacts with heparan sulfate proteoglycans, potentially affecting axonal guidance [26]. zNT-7, isolated from zebrafish, shows binding affinity to rat TrkA and human p75 receptors [27].

### 1.4. Neurotrophins and p75NTR Receptors

While Trk receptors interact with specific neurotrophins to activate neuronal survival and differentiation pathways, the p75NTR receptor, a TNFR superfamily member, binds all neurotrophins and triggers various cellular responses, such as survival, apoptosis, and neurite outgrowth [28,29]. Although Trk and p75 receptors can function independently [30], when they are co-expressed in the nervous system, it allows p75 to increase Trk’s binding affinity and specificity for neurotrophins, thereby promoting survival signaling [12,31]. In contrast, neuronal death can result from p75 receptor activation in the absence of Trk signaling [32]. The p75NTR receptor has a single transmembrane domain, a short intracellular death domain, and a large extracellular domain with four cysteine-rich regions for ligand binding and receptor dimerization, which is necessary for adaptor protein interaction, receptor activation, and apoptotic and inflammation signaling, among others [33]. Also, all pro-neurotrophins show a high affinity for sortilin, which is necessary for the pro-NT-p75NTR activation of apoptosis [34]. Research indicates that ProBDNF interaction with p75NTR and sortillin is essential to trigger neuronal apoptosis and activate the caspase pathway [35,36,37,38]. In addition, the prodomain of BDNF can exclusively bind to sortillin receptors [39]. Furthermore, p75NTR has dual functions. Thus, when activated by nerve growth factor (NGF), it promotes survival, while when activated by brain-derived neurotrophic factor (BDNF), it induces apoptosis [40]. Also, it recruits TRAF6, which influences cell motility, inflammation, and survival by regulating Rho GTPases, activating the NF-κB signaling pathway, and stimulating ceramide synthesis [14]. p75NTR can interact with Nogo receptor, MAG, and OMgp, suggesting inhibitory activities in neuronal outgrowth [41].

**Table 1:** Neurotrophins and their receptors.

Neurotrophin	Receptors
NGF	TrkA [18,19,20]
	p75NTR [29,33]
BDNF	TrkB [21]
	p75NTR [29]
	TrkBT1 [25]
NT-3	TrkC [22,23]
	TrkA and TrkB [24]
	p75NTR [29]
	TrkbT1 [25]
NT-4/5	TrkB [21]
	TrkA [24]
	p75NTR [29]
	TrkbT1 [25]
NT-6	Heparan sulfate proteoglycans [26]
NT-7	Rat TrkA and human p75NTR [27]
proNGF	Sortillin/p75NTR [29,34,35]
proBDNF	
pro NT-3	
pro NT4/5	

### 1.5. Regulation of Neuronal Apoptosis by Neurotrophins

**Figure 1:**
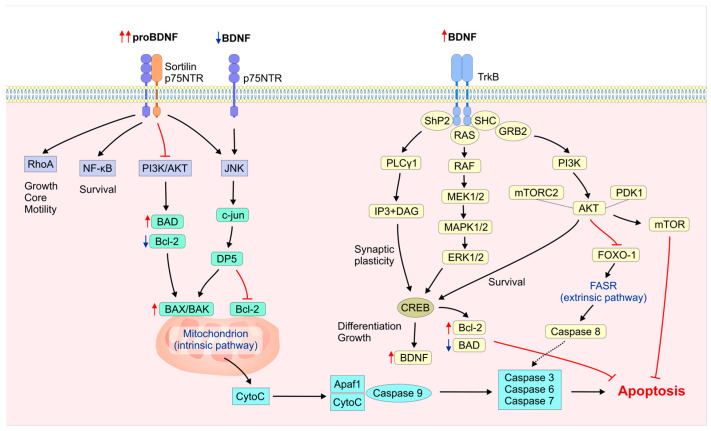
BDNF and proBDNF pathways in apoptotic signaling regulation.

Neurotrophins and their receptors are critical in neuronal survival and apoptosis through the activation of complex signaling pathways. The functional balance of the nervous system requires neuronal proliferation, differentiation, migration, survival, and death [42]. Neuronal apoptosis can be seen as a tightly controlled self-destruction process that acts to keep the balance through intrinsic and extrinsic apoptotic pathways (Figure 1).

Damage to DNA and oxidative stress are examples of internal stress signals that can activate the intrinsic mitochondrial apoptotic pathway. This may cause the activation of proteins that promote cell death, such as Bak and Bax, which cause mitochondrial membrane permeability to increase, leading to the release of cytochrome c, the activation of the caspase cascade, and ultimately cell death (Figure 1) [43]. The BCL-2 protein family regulates the mitochondrial apoptotic pathway that maintains internal cell balance, even under stressful conditions. The coordination of pro- and anti-apoptotic proteins determines cell fate. It has been shown that the dysregulation of the BCL-2 protein family is linked to neuronal degeneration [44], and the pro-apoptotic BCL-2 family protein BAX causes neuronal death during neuronal development or after NGF removal [45]. Extrinsic apoptosis is initiated by death receptors on the neural surface which bind to ligands such as Fas and TNF to activate caspase cascades, resulting in apoptosis [43]. One study found that BDNF/p75NTR regulated FAS-induced apoptosis in human neuroblastoma cells, underscoring this regulatory network’s importance for neuronal health [46]. Dopaminergic neuronal BDNF deficiency also triggered neuronal apoptosis via activating the Fas death receptor pathway and caspase-8 cascade [47].

Apoptosis is necessary for neuronal growth because it removes excess or misplaced neurons to improve neural networks [48,49]. It contributes to the accurate development of these neural circuits, but excessive or inappropriate apoptosis can lead to neurodegenerative diseases like Alzheimer’s, Parkinson’s, and Huntington’s, psychiatric disorders, stroke, and traumatic brain injury [50,51]. BDNF and its receptor TrkB are essential for neuronal survival and plasticity; their deficiency can lead to detrimental consequences, ultimately resulting in neuronal death. Conversely, proBDNF and its receptor p75NTR are associated with apoptosis (Figure 1) [52]. The modulation of BDNF and proBDNF relationships is critical for sustaining normal neuronal function; it affects neuronal populations throughout development, injury responses, and neurodegenerative disorders via intricate interactions.

## 2. Molecular Mechanisms of BDNF and proBDNF in Neuronal Apoptosis Regulation

Understanding the molecular mechanisms behind the reciprocal control of apoptosis by BDNF and its precursor, proBDNF, is essential for developing treatment for neurological diseases defined by excessive death. Numerous studies have clarified the mechanisms by which proBDNF and BDNF affect neuronal health and death.

### 2.1. BDNF/proBDNF Origin and Synthesis

BDNF mRNA and proteins are expressed in all of the brain’s regions, particularly in the hippocampus and cerebral cortex, and play crucial neurophysiological roles in the brain [53,54,55]. The functions of BDNF and proBDNF vary depending on brain development stage and cell type. After membrane depolarization, pro-BDNF and BDNF are released in neuronal cells, maintaining a dynamic balance between different BNDF isoforms [56]. BDNF/proBDNF balance fluctuates across stages of brain development and regions, with pro-BDNF levels increasing during early postnatal periods and BDNF being predominant in adulthood [57]. Pro-BDNF is essential for brain function development, while BDNF is vital for adult functions like synaptic plasticity, glial cell creation, neuroprotection, memory, cognition, and social behaviors [39,58].

The human gene for BDNF, located on chromosome 11p14.1, has a complex structure consisting of 11 exons upstream and nine functional promoters. Exon IV encodes the protein’s codon sequence. The eight upstream exons regulate the transcription of several BDNF transcripts, resulting in the production of the same protein in specific areas of neuronal and non-neuronal tissues [59].

The translation process converts BDNF mRNA into pre-proBDNF, a 32 kDa precursor protein with a prodomain for folding and secretion. This occurs in the endoplasmic reticulum, where it is glycosylated and folded with chaperone proteins and then cleaved into pro-BDNF. ProBDNF is secreted through the Golgi apparatus and packed into secretory vesicles. It is cleaved by proteolytic enzymes, primarily furin and proconvertases, to form the mature 14 kDa BDNF protein. Other enzymes, including plasmin and matrix metalloproteinases (MMPs), can also cleave pro-BDNF extracellularly [60].

BDNF and pro-BDNF aggregate into vesicles and have different functions when released from cells. BDNF activates signaling pathways that support neuronal survival, differentiation, synaptic plasticity, and cognition via the TrkB receptor. ProBDNF activates JNK, Ras/Rho, and NF-kB via p75NTR and sortillin. These pathways control neuron growth, survival, and apoptosis [39,59,61,62,63]. Conversely, BDNF’s p75NTR activation may also cause neuronal apoptosis [37,64].

### 2.2. BDNF/TrkB Activation

**Figure 2:**
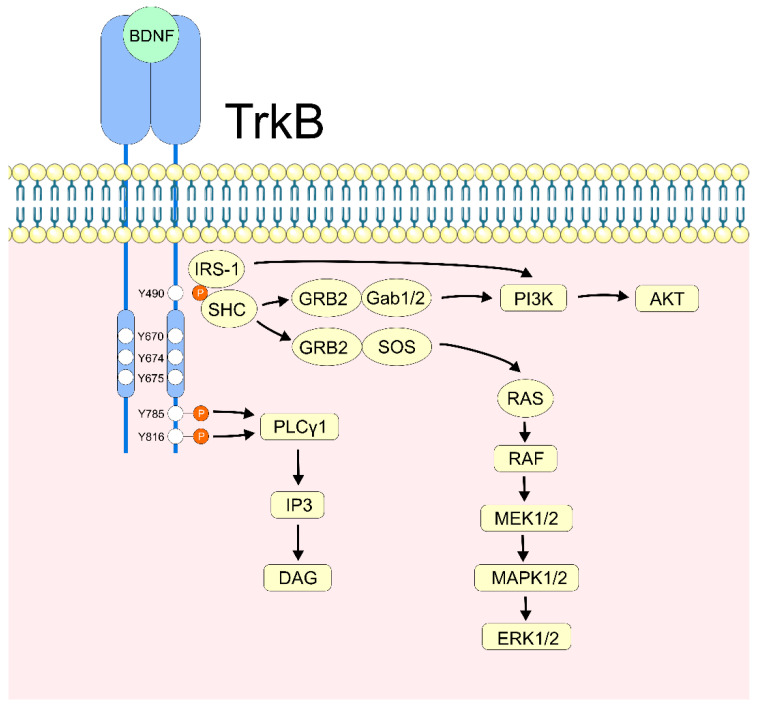
BDNF/TrkB activation and signaling pathways.

The mature BDNF homodimer dimerizes the TrkB receptor and activates intracellular kinase domains via a cysteine knot motif. Receptor activation is modulated by tyrosine phosphorylation—of Y670, Y674, and Y675—in TrkB’s cytoplasmic domains (Figure 2). Tyrosine residues Y490 and Y785 activate downstream signaling by interacting with adaptor proteins with phosphotyrosine-binding (PTB) or src-homology-2 (SH-2) motifs [16,65]. Phosphorylation on residues Y785 or Y816 activates the PLCγ/DAG/IP3 signaling pathway, while pY490 recruits and phosphorylates Shc, activating the Grb2/SOS complex and Ras/Raf/MEK/ERK pathways or the Grb2/Gab1/2 complex and the PI3K/Akt pathway [66,67]. PI3K signaling can also be activated directly by pY490 binding IRS-1 [16] (Figure 2).

### 2.3. BDNF’s Neuronal Apoptotic Regulation via PI3K/Akt

The PI3K/Akt signaling pathway is a critical mechanism that regulates cell functions, including growth, division, metabolic homeostasis, and neuron survival [68,69]. BDNF-TrkB interaction activates PI3K, which phosphorylates PIP2 to make PIP3, a docking site for Akt and PDK1. Akt is weakly activated by PDK1 through phosphorylation at threonine 308, whereas mTORC achieves full activation at serine 473. Active Akt phosphorylates downstream substrates including mTOR, GSK3β, NFkB, FOXO transcription factors, Bcl-2 family members, and caspases to regulate anti-apoptotic and neuronal survival (Figure 1) [61,70].

Multiple studies have highlighted the protective role of BDNF/Akt signaling in neuronal health. Given the foregoing, a study found that BDNF can reverse high-glucose-induced apoptosis in hippocampal neurons by upregulating TrkB, Akt, Syn, Arc, and CREB, which enhance synaptic plasticity [71]. In addition, BDNF/TrkB/Akt signaling prevents hypoxia/reoxygenation-induced apoptosis in human cortical neurons and astrocytes while maintaining mitochondrial membrane potential [72]. In addition, BDNF activation of the Akt pathway increased neuroblastoma cell proliferation and decreased ropicavine-induced apoptosis by increasing Bcl-2 and PCNA expression and decreasing Bax and cleaved caspase-3 expression [73]. Furthermore, research indicated that BDNF protected neuroblastoma cells from Aβ25-35-induced apoptosis by activating PI3K/AKT and phosphorylating GSK3β [74]. More intriguingly, Sheikh and colleagues found that BDNF dysregulation weakened the BDNF-Akt-Bcl2 anti-apoptotic signaling pathway by decreasing Akt activation. Decreased Bcl-2 and increased p53 expressions led to neuronal death and the genesis of autism through modified apoptotic pathways [75]. A separate study demonstrated that BDNF reduced striatal cell death via modifying NMDAR activity and activating the Akt pathway, which increased Bcl-2 and decreased Bax and caspase-3 [76]. According to a recent study, BDNF also reduced neuronal death in Parkinson’s disease via activating STAT3/PI3K/AKT/mTOR autophagy [77].

### 2.4. BDNF’s Neuronal Apoptotic Regulation via MAPK/ERK and MAPK/p38

BDNF/TrkB activation promotes the MAPK/ERK signaling pathway, which regulates apoptotic proteins and neuronal survival, growth, and plasticity [61,78]. The MAPK/ERK pathway is initiated by the activation of Ras, which phosphorylates Raf and MEK, activating ERK1/2. This activation inhibits pro-apoptotic proteins like Bad and increases the transcription of Bcl-2 anti-apoptotic proteins through transcription factors like CREB, Elk-1, and AP-1 (Figure 1) [11,79]. It has been demonstrated that BDNF is able to decrease apoptosis in rat cortical neurons by stimulating ERK1/2 and PDK1, which then activate RSK1/2 and Elk-1, facilitating neuronal survival [80].

Stressors such as oxidative stress, inflammatory cytokines, or BDNF induce the p38 MAPK pathway in certain neurons, leading to the activation of inflammation-related gene expression and the phosphorylation of MAPKAPK2 and ATF2 [81]. BDNF/p38MAPK activation plays a multifunctional role in neuronal survival, having pro-apoptotic and pro-survival actions depending on the context. For example, one report claimed that BDNF increases the neurite outgrowth of neonatal cochlear spiral ganglion explants through the MAPK/p38 and PI3K/Akt pathways [82]. In addition, BDNF decreased TNF and β-amyloid peptide-induced cytotoxicity in cholinergic neurons by downregulating JNK activation through p38 MAPK, indicating possible therapeutic uses for Alzheimer’s disease [83]. On the other hand, BDNF was also implicated in promoting NO-induced apoptosis in embryonic cortical neurons through the activation of p38 MAPK and MAPK/ERK pathways [78]. Indeed, these results emphasize the necessity for further studies to elucidate BDNF’s dual function in apoptosis and neuronal survival.

### 2.5. Regulation of Neuronal Apoptosis via BDNF/Akt and BDNF/ERK Cross-Talk

Numerous studies have highlighted the interaction between BDNF-activated PI3K/Akt and MAPK/ERK pathways in regulating neuronal apoptosis. Research indicated that BDNF inhibited glutamate-induced apoptosis in hippocampal neurons by activating the Akt and ERK pathways, which subsequently inhibited caspase-3 and enhanced Bcl-2 expression. Moreover, a synergistic neuroprotective mechanism was suggested by the fact that blocking one pathway reduced the anti-apoptotic effect of the other [84]. Additionally, BDNF/ERK and BDNF/Akt pathways have been shown to protect cortical neurons from 3-NP neurotoxicity, autophagy, oxidative stress, and mitochondrial dysfunction by inhibiting Bim and caspase-3 activation and activating mTOR/c-jun signaling [85,86]. BDNF also activated ERK and Akt pathways to reduce FOXO3a-induced Bim levels, thereby promoting neuronal survival in retinoic acid-induced neuroblastoma cell apoptosis [87]. Furthermore, BDNF/TrkB-T1 signaling in astrocytes activated ERK and Akt pathways that prevented serum deprivation-induced apoptosis and oxidative stress and enhanced neuroprotection by inhibiting caspase-3 and p53 activation [88]. Another study discovered that BDNF and TrkB overexpression could prevent Huntington’s disease-mediated apoptosis in striatal cells by delaying caspase-3 activation via Akt and ERK survival pathways [89].

### 2.6. BDNF/PLCγ Signaling in Neuronal Survival and Apoptosis

When BDNF binds to TrkB, it triggers PLCγ1, which cleaves PIP2 into DAG and IP3, triggering crucial neuronal survival pathways. IP3 plays a role in the mobilization of Ca^2+^ into the cytoplasm, while DAG is required for activating protein kinase C (Figure 1) [90]. BDNF/PLCγ/Ca^2+^ signaling controls axonal signaling and synaptic plasticity [90,91,92]. While the activation of MAPK and PI3K pathways by BDNF/TrkB has been widely studied for its function in neuronal survival, the PLCγ pathway has recently been found to be an important regulator of the anti-apoptotic actions of BDNF [93]. In fact, BDNF suppresses Aβ-induced neuronal apoptosis by inhibiting the activation of caspase-3 and calpain, potentially through the PLCγ pathway [94]. Not much research has been conducted on BDNF’s involvement in PLCγ pathway activation in neuronal apoptosis; however, PLCγ’s pro-apoptotic effects are acknowledged in certain signaling environments [95].

### 2.7. The Role of ProBDNF in Neuronal Apoptosis

ProBDNF is capable of activating downstream signaling pathways, such as RhoA, JNK, and NF-κB, by binding to receptors such as p75NTR and sortilin (Figure 1). This process results in neuronal apoptosis and other neuronal–immunity–endocrine functions [96]. Furthermore, the activation of the proBDNF/FSTL4 pathway could induce neuronal apoptosis; however, the downstream signaling of this pathway has remained uncertain [97]. In this context, a study found that proBDNF increased post-traumatic depression (PSD) by binding to the p75NTR receptor, activating RhoA/JNK signaling, and increasing cytochrome c and caspase-3 expression, inhibiting nerve synapse rebuilding and accelerating PSD [98]. Moreover, in neonatal rats, proBDNF caused sensory neuron apoptosis after sciatic nerve injury. Sortilin or proBDNF neutralization improved neuron survival after injury. Furthermore, after blocking proBDNF, sensory neurons in the contralateral DRG were more likely to survive and proliferate, demonstrating proBDNF’s dual role in neuronal differentiation and maturation [99].

### 2.8. The Role of BDNF/proBDNF Interplay in Neuronal Survival and Apoptosis

Numerous studies have demonstrated the complex mechanisms underlying the BDNF/proBDNF interplay in mediating neuronal apoptotic or survival mechanisms. One study examined the interplay between proBDNF and BDNF in neuronal apoptotic mechanisms during status epilepticus (SE) and concluded that BDNF was essential for promoting neuronal survival after SE, whereas proBDNF was linked to neuronal death. Furthermore, the depletion of BDNF resulted in an increase in proBDNF signaling via the p75NTR receptor, which was associated with apoptosis. This suggested that the absence of BDNF tipped the balance in favor of proBDNF’s detrimental effects [52]. Another study found that proBDNF induced mitochondrial apoptosis of sensory neurons and satellite glial cells (SGCs) after sciatic nerve transection by modulating BDNF-TrkB and proBDNF-p75NTR/sortilin signaling pathways. Anti-proBDNF serum injection effectively prevented the activation of SGCs and promoted their proliferation, thereby promoting neuroprotection [100]. In another study, proBDNF activated p75NTR and sortilin to cause neuronal apoptosis, but mature BDNF promoted survival and differentiation via TrkB in a sympathetic neuron culture model. This interaction was suggestive of proBDNF’s role in neuronal death-related neurological disorders [36]. Additionally, in a separate study, BDNF and proBDNF regulated cerebellar granule neuron apoptosis differently. BDNF activated TrkB, ERKs, and Akt and inhibited caspase-3, while proBDNF activated p75NTR, Rac-GTPase, JNK, and caspase-3 to induce apoptosis [101].

Although a series of studies emphasized the importance of the duality of proBDNF and BDNF in regulating complex mechanisms of neuronal survival and apoptosis, a comparative analysis of proBDNF/BDNF interplay across neurodegenerative disorders is essential to further enrich analytical depth. In Alzheimer’s disease (AD), Parkinson’s disease (PD), and Huntington’s disease (HD), the modulation of neuronal apoptosis and survival through the BDNF/proBDNF axis shows both conserved and context-specific patterns. Nevertheless, the activation of mitochondrial apoptosis by an elevated proBDNF/BDNF ratio is a shared feature of all diseases (Figure 1). In AD models, a notable trend is observed with increased proBDNF levels and decreased BDNF levels, which are associated with synaptic loss and degeneration of the hippocampus. This relationship is particularly influenced by proBDNF/p75NTR/sortilin apoptotic mitochondrial signaling [102]. In PD models, mitochondrial apoptotic activation is characterized by decreased TrkB and PI3K/Akt and MAPK/ERK activation, as well as elevated Bax/Bcl-2 ratios. BDNF therapy has shown promise in restoring dopaminergic neuron survival despite limits of the blood–brain barrier [103]. In HD models, an increased proBDNF/BDNF ratio significantly contributes to mitochondrial dysfunction and cell death, while reduced BDNF availability exacerbates neurodegeneration in striatal neurons. TrkB overexpression significantly delays caspase-3 activation and promotes cell survival by activating the PI3K/Akt and ERK signaling pathways [104].

The varying effects of proBDNF/BDNF interplay on neuronal apoptosis and survival under different conditions can be attributed to variations in receptor expression profiles (such as truncated versus full-length TrkB), cell-type-specific susceptibility, or the expression of neurotrophin-related enzymes and cofactors that are influenced by developmental stages. For instance, in PD, dopamine induces the clustering of TrkB receptors, whereas in AD, there is a downregulation of TrkB. This contrast highlights the unique regulatory environmental milieu associated with each cell type. The inclusion of these comparative analyses can reveal overlapping pathways and emphasize differences that are crucial for the development of neurotrophin-based therapies aimed at specific disorders.

## 3. Factors Regulating BDNF/proBDNF Balance

The balance between mature BDNF and proBDNF is tightly regulated by a complex interplay of factors, including proteolytic cleavage, gene expression regulation, receptor interactions, phosphorylation mechanisms, intracellular trafficking, neuronal activity, and specific environmental stimuli. Understanding the factors that regulate this delicate equilibrium provides a crucial avenue for therapeutic interventions targeting neurological disorders.

### 3.1. BDNF Gene Transcription

The human BDNF gene consists of eleven exons and nine functional promoters that collaboratively produce various transcripts. The varied utilization of promoters and alternative splicing results in the generation of at least 18 distinct mRNA transcripts. These transcripts demonstrate context-dependent BDNF expression through unique brain expression patterns [59]. For example, the prefrontal cortex of patients with Alzheimer’s disease exhibited a decrease in BDNF transcripts containing exons I, II, and IV [105]. There are various levels of regulation, including transcription, post-transcription, and epigenetics. BDNF transcription is controlled by diverse stimuli, including exercise, stress, neural activity, and nutrition [106]. Following transcription, BDNF mRNA undergoes differential splicing and is transported dendritically to facilitate the local translation necessary for synaptic plasticity [107]. Changes in these factors can alter the overall production of pre-proBDNF. Increased neuronal activity, particularly synaptic transmission, is a potent stimulus for BDNF gene expression. This activity-dependent regulation is mediated by calcium influx and the activation of transcription factors like CREB (cAMP response element-binding protein) [108]. Epigenetic regulation occurs through DNA methylation, histone modification, and the expression patterns of non-coding RNAs (mRNAs). mRNAs play an important role in the expression and signaling impairment of BDNF in several neurodegenerative disorders [109], while epigenetic modifications of BDNF, particularly histone modifications of the BDNF gene, are closely linked to the pathophysiology of Huntington’s and Alzheimer’s diseases [110]. Currently, there are limited studies examining the epigenetic mechanisms affecting BDNF expression in Parkinson’s disease pathology [111].

One study found that the intricate regulation of BDNF gene expression in cortical and hippocampal neurons is facilitated by CRTC1 and CBP coactivators as well as CREB transcription factors. CREB plays a crucial role in initial BDNF mRNA activation, which diminishes as long-term neuronal activity increases. The identification of novel transcription factors like SATB2, FOXP1, RAI1, TCF4, and BCL11A, as potential regulators of BDNF expression, suggests the necessity of targeted therapies for neurodegenerative disorders characterized by defective BDNF signaling [112]. Moreover, gene polymorphism may potentially influence the level of BDNF in the blood, brain, and peripheral tissues. Another study found that BDNF polymorphisms, particularly the Val66Met variant, were linked to a higher proBDNF/BDNF ratio, which negatively impacted neural plasticity and Default Mode Network activity in individuals with normal cognitive function. Additionally, it emphasized the correlation between increased BDNF levels and improved DMN connection, highlighting the role of hereditary factors in controlling the proBDNF/BDNF balance, which may be essential for the diagnosis and treatment of Alzheimer’s disease [113]. Moreover, the Val66Met variant of the BDNF gene is associated with elevated proBDNF levels, which lead to decreased dendritic distribution, impaired BDNF transport to secretory granules, and reduced activity-dependent BDNF secretion, contributing to PD pathology [114]. In addition, research has suggested that the Val66Met BDNF gene polymorphism is linked to lower blood BDNF levels and an increased risk of early-onset Parkinson’s disease (PD) [115].

The expression of BDNF in neurons is regulated by the MEF2 group of transcription factors, MEF2A-D, which have been associated with Alzheimer’s and Parkinson’s diseases [116]. BDNF exon IV expression was found to be upregulated by MEF2C and BDNF exon I expression downregulated by MEF2D, suggesting highly complex regulation [117]. The effect of MEF2 proteins is illustrated by BDNF expression in hippocampal neurons dependent on a −4.8 kb enhancer region [118]. Recent research suggests that MEF2D may serve as a potential target for pharmacological intervention in neurodegenerative illnesses, specifically Parkinson’s disease [119,120].

Physical exercise significantly enhances BDNF production within the human brain, enabling neurogenesis and cognitive improvement. Research shows that exercise is beneficial in increasing the levels of BDNF mRNA and protein in the hippocampus and brain regions [121]. As an example, voluntarily running mice exhibited elevated levels of BDNF expression within the II/III and V cortical layers of the brain versus sedentary controls. Eliminating BDNF function destroys the cognitive responses of exercise, indicating its critical role in brain plasticity [122]. Furthermore, exercise training has also been found to increase BDNF levels and improve motor symptoms in patients with Parkinson’s, though controversies exist regarding the best exercise intensity and pathways for elevated circulating BDNF levels in this case [123].

The expression of BDNF is also regulated by hormones [124]. A recent study indicated that estradiol (E2) enhanced the expression of BDNF in the hippocampus by promoting the binding of estrogen receptors and coactivators to the BDNF promoter. Additionally, under basal conditions, the long non-coding RNA HOTAIR represses BDNF expression by recruiting silencing complexes. In addition, HOTAIR knockdown alleviated this repression, leading to increased BDNF levels. The findings elucidate the complex interplay between E2 and HOTAIR in modulating BDNF gene expression, which is crucial for neuronal growth and maintenance [125].

### 3.2. proBDNF/BDNF Proteolytic Cleavage

Enzymes such as furin and intracellular proprotein proteases, along with extracellular matrix metalloproteinases and plasmin, undergo proteolytic cleavage to convert pro-BDNF into mature BDNF (mBDNF) [126]. Cai et al. (2020) indicated that Alzheimer’s disease model mice exhibited reduced plasmin levels in the hippocampus [127]. Neuronal activity drives the cleavage of proBDNF into BDNF, which is critically important for neuronal function. However, the aberrant cleavage of pro-BDNF may lead to neurotoxic effects. Plasminogen activator inhibitor-1 (PAI-1) controls the plasmin-dependent processing of proBDNF in the brain [128]. One study demonstrated that in models of AD, increased amounts of Aβ soluble protein caused PAI-1 levels to rise, which in turn inhibited pro-BDNF cleavage and raised the proBNDF/BNDF ratio. In contrast, PAI-1 inhibition with TM5A15 reduced Aβ levels and increased BDNF levels, improving memory performance in AD mouse models [129].

Apolipoprotein E4 (ApoE4) has been identified as a significant genetic risk factor for late-onset Alzheimer’s disease, as it has been associated with reduced BDNF levels [130] and inhibited BDNF mRNA expression [131]. Research indicated that different ApoE isoforms, particularly ApoE2, enhanced the conversion of proBDNF to BDNF, thereby increasing BDNF levels. In contrast, ApoE4 resulted in minimal BDNF production from proBDNF, indicating that ApoE4/BDNF-related defects in proBDNF processing might lead to AD pathology [132].

### 3.3. TrkB and p75NTR/Sortillin Receptor Expression

TrkB in humans is mainly expressed in three splice variants. The most prominent isoform, full-length TrkB (TrkB-FL), consists of leucine-rich motifs, cysteine-rich domains and immunoglobulin-like domains in the extracellular region, and an intracellular tyrosine kinase domain, which rapidly transmits the effects of neurotrophin binding to the downstream molecules. Alternative splicing of the primary transcript produces variants as truncated TrkB isoforms (T1 and T-shc in humans; T1 and T2 in rats) that lack the intracellular catalytic domain and act like the dominant negative regulators of TrkB-FL [133,134]. TrkB-T1 is predominantly expressed in the brain but also detected in other tissues like the heart, kidney, and pancreas. Another isoform, TrkB-T-Shc, is mostly expressed in the brain [135]. TrkB-FL stimulates pro-survival signaling pathways like PI3K/Akt, MAPK/ERK, and PLCγ in response to BDNF binding, which promotes synaptic plasticity, neuronal differentiation, and anti-apoptotic effects (Figure 1). The dysregulation of TrkB, especially the reduced expression or an increased ratio of truncated to full-length isoforms, may shift the balance between neuroprotective and neurotoxic effects. This phenomenon has been observed in neurodegenerative diseases, including AD and PD, and results in reduced trophic support and heightened vulnerability of neurons [136,137,138]. Specifically, one study revealed a significant change in the distribution of TrkB-FL and TrkB-T1 in the SNpc and striatum of individuals with Parkinson’s disease (PD). Additionally, there was a reduction in dendritic catalytic receptor isoforms, suggesting a decline in synaptic function [139]. Moreover, either the inhibition of BDNF expression or TrkB deficiency resulted in the death of SNpc dopaminergic neurons [140]. The absence of BDNF/TrkB survival signals via the PI3K/Akt pathway in SN dopaminergic neurons has been associated with increased caspase-3 activity and the initiation of apoptosis, which may contribute to the development of PD [141]. TrkB signaling is essential in AD, as multiple studies have demonstrated that the expression levels of BDNF and the TrkB receptor are significantly reduced in patients with AD [142]. Furthermore, AD has been associated with increased production of pro-apoptotic proteins as well as enhanced activation of the caspase-9-mediated mitochondrial apoptosis pathway [143]. Mitochondrial dysfunction has long been regarded as a crucial element in the onset and progression of AD. Consequently, a deficiency of BDNF/TrkB results in the inhibition of PI3K/Akt survival signaling and the activation of Bax/caspase-9 mitochondrial apoptosis, which are key factors in the pathophysiology of Alzheimer’s disease (Figure 1). Therefore, enhanced TrkB receptor-mediated PI3K/AKT activation may be crucial for reducing AD-related neuron loss and cognitive impairment.

p75NTR is widely expressed in the developing brain [144], but during adulthood, it becomes refined to specific brain regions [145] including cholinergic neurons of the basal forebrain and neurons and astrocytes of hippocampal CA1, amongst others [146,147]. The pan-neurotrophin receptor, p75NTR, promotes distinct signaling pathways in cells that in most cases oppose but sometimes coordinate with TrkB-promoted pathways [148]. P75NTR may also modulate TrkB actions through its capability to influence receptor conformation, resulting in altered TrkB specificity and affinity to neurotrophins under normal and pathological conditions [16]. Unlike BDNF, which binds to TrkB, proBDNF preferentially binds to p75NTR. It frequently forms a complex with the co-receptor sortilin, which activates pro-apoptotic signaling cascades such as the RhoA, JNK, and caspase-3 pathways (Figure 1). Increased expression of p75NTR and sortillin has been observed in response to stress or injury, correlating with increased apoptosis, particularly in models of degeneration involving hippocampal, cortical, and dopaminergic neurons [149]. In fact, a study demonstrated a two-fold increase in p75NTR receptor expression in the hippocampal membranes of patients with AD [150]. Studies conducted on p75NTR-deficient mice suggest that Aβ can directly activate p75NTR-mediated cell death through the selection of downstream death effectors. This finding links Aβ/p75NTR apoptotic activation to the pathology of Alzheimer’s disease [151]. Moreover, increased p75NTR expression was associated with the severity of dopaminergic neuronal injury through the apoptotic activation of JNK, caspase-3, nuclear factor kappa B (NF-κB), and RhoA pathways, implicating p75NTR signaling in the pathogenesis of PD [152]. The proBDNF/p75NTR axis can override TrkB-mediated survival signals, promoting apoptotic signals instead, particularly when BDNF availability is low or when the extracellular environment is more favorable to the accumulation of precursor proBDNF rather than the activity of mature BDNF neurotrophin [153].

The expression levels of TrkB and p75NTR/sortillin receptors can impact BDNF and proBDNF responses to shift the balance toward neuronal survival or apoptosis. One study investigated dopamine’s role in regulating the sensitivity of striatal medium spiny projection neurons (SPNs) to brain-derived neurotrophic factor (BDNF). The study established that dopamine stimulation increased TrkB cell surface translocation and expression and raised BDNF sensitivity. Conversely, dopamine depletion reduced BDNF responsiveness and formed intracellular clusters of TrkB. The TrkB clusters bonded with sortilin-related VPS10 domain-containing receptor 2 (SORCS-2) and were likely to contribute to impaired motor function in PD [154]. Alongside this, the study detected the early onset of AD with enhanced brain atrophy and an increased proBNDF/BDNF ratio in middle-aged wild-type mice. Increased proBDNF, especially in the hippocampus, may result in susceptibility to Alzheimer’s. In addition, the study detected a correlation between the proBNF/BDNF ratio and the loss of dendritic spines across ages, which is related to Alzheimer’s development, suggesting that the proBDNF/BDNF ratio may serve as a biomarker and therapeutic target for AD treatment. The imbalance’s causes are unknown, but truncated TrkB expression increased with age in the brain stem and inhibited full-length TrkB pro-survival signaling. However, full-length TrkB and p75NTR decreased in the hippocampus but remained constant elsewhere in the brain [155].

All considered, the interplay between TrkB and p75NTR signaling pathways sets the molecular equilibrium that counterbalances the bifunctional activities of BDNF and proBDNF—neuroprotection or neurodegeneration. Clarifying and targeting the dynamics of these receptors hold significant therapeutic potential for regulating cell survival in various neuropathological conditions.

## 4. The Clinical Implications of Dysregulated BDNF/proBDNF Interplay in Apoptotic-Related Neurodegenerative Disorders

Neurodegenerative diseases are neurological disorders characterized by progressive neuron loss and abnormal protein deposition in specific brain regions, causing physical and cognitive disability. These diseases affect around 15% of the global population and are increasing in incidence [156]. To address the challenge of maintaining neurological care, biomedical disciplines are focusing on investigating the interrelationships of the underlying pathophysiological background. The role of neurotrophin system elements in these processes is crucial in addressing symptomatology and preventing one of the biggest medical threats of the new age. The global Alzheimer’s disease population is projected to double by 2060, highlighting the significant medical, societal, and financial implications of each scientific breakthrough in this field [157].

### 4.1. BDNF/proBDNF in the Apoptotic Mechanisms Associated with Alzheimer’s Disease

Over the past decade, multiple studies have employed stereotaxic methods to induce Alzheimer’s disease in rats, with the results summarized in Table 2. Du and colleagues [158] investigated neurotrophin signaling changes in rat models of Alzheimer’s disease, specifically through the intrahippocampal administration of Aβ25–35, and revealed pro-apoptotic effects including increased Bax and caspase-3 and decreased Bcl-2 expression. Antioxidant supplementation with Angelica sinensis polysaccharide effectively mitigated the molecular mechanisms associated with cognitive impairment, as demonstrated in Table 2. Zhou and Xu [159] applied a similar model for AD induction, which resulted in a decline in BDNF, NT-3, and β-NGF. They found that AD behavioral manifestations coincided with pro-apoptotic events in hippocampal tissue including Bax and caspase-3 increase and Bcl-2 decrease. They confirmed the significance of brain-specific angiogenesis inhibitor-1 (BAI1), which inhibited astrocyte activation via the STAT3/EZH2 pathway and promoted BDNF secretion, thereby reducing neuronal apoptosis. Various studies have established evidence of microRNAs (miRNAs) regulating BDNF expression [160] and playing important roles in neurophysiological processes by regulating cell proliferation, differentiation, and apoptosis. In a stereotaxic AD rat model, Ren and colleagues [161] investigated the pathophysiological mechanism of AD by administering Aβl–42 peptide to primary cerebral cortex neurons and PC12 cells. The study found decreased miR-125b-5p expression and increased lncRNA BDNF antisense (BDNF-AS) expression in AD models, leading to cell apoptosis, inflammation, and inflammatory pathway-related proteins. The knockdown of BDNF-AS or intervention with miR-125b-5p reduced these effects, suggesting that the miR-125b-5p/BDNF-AS ratio may serve as a biomarker and therapeutic target for AD treatment. Wang and other researchers [162] studied the effects of miR-22 on an experimental AD rat model, confirming its benefits by restoring BDNF and expressing the Bcl-2 anti-apoptotic marker and by decreasing Bax expression in hippocampal tissue samples. Babaei and coworkers [163] employed a similar method for AD induction using the Aβl-42 peptide, which resulted in cognitive impairment, a decline in BDNF, NGF, and Bcl-2 levels, and an increase in Bax and caspase-3 expression (Table 2). The therapeutic approach, using mesenchymal stem cells from adipose tissue in the brain tissue (in situ), was statistically sufficient to prevent AD manifestations and apoptosis, but only after preconditioning with dimethyl fumarate.

Cui and other researchers [164] conducted a study using neural stem cell transplantation to compete with markers in an experimental AD model (Aβ1–40 to CA1 hippocampal region). The results showed improved cognitive functions, the restoration of BDNF levels, and the amelioration of apoptosis, including a decline in Bax and caspase-3 activation in the affected region, as well as a decrease in pro-inflammatory cytokines. Hu and coworkers [165] found that BDNF-transduced human umbilical cord mesenchymal stem cell-derived cholinergic-like neurons (hUC-MSCs) improved spatial learning and memory in rat models of AD. The treatment also normalized BDNF levels and decreased apoptotic markers in the hippocampus, providing a possible therapeutic strategy for Alzheimer’s disease. Furthermore, Gu and other researchers [166] found that the Aβl-42 peptide caused neurodegenerative changes in a stereotaxic rat model of AD, including cognitive impairment, increased pro-apoptotic markers (Bax, caspase-3 and -9), and decreased Bcl-2 and BDNF levels. However, the effects of Alzheimer’s disease were prevented by extracts of the flavonoid-rich plant Cynomorium songaricum, indicating potential therapeutic benefits. In addition, the efficacy of a stereotaxic approach was supported by a study that showed the activation of the mitochondrial apoptotic pathway associated with Alzheimer’s disease and a decrease in monoamines in brain tissue. Over the course of six weeks, carbenoxolone therapy demonstrated anti-inflammatory and anti-apoptotic effects, maintaining mitochondrial activity and inhibiting caspase-9/3 activation [167]. Additionally, research by Postu and colleagues [168] indicated that a methanolic extract of Lactuca capensis elevated BDNF levels, inhibited apoptosis, and improved behavioral changes as well as biochemical markers associated with AD. Wang’s research group tested a therapeutic strategy for ameliorating neurochemical changes in Alzheimer’s disease (AD) by performing two protocols after surgery: exercise rehabilitation and supplementation with astragaloside. Both protocols showed beneficial effects by reversing AD hallmarks, such as a decline in BDNF and TrkB, hippocampal and cortical apoptosis, and cognitive and motor impairment [169]. Another study used Icariside II, an active component from Epimedium brevicornum, for the treatment of stereotaxically induced AD in rats and P12 cell cultures. The supplementation effectively attenuated cognitive impairment and reversed the decline in BDNF and TrkB. It also compensated for alterations in apoptotic equilibrium accompanied by AD, such as an increase in Bax and active caspase-3 and a decrease in Bcl-2 [170]. Che and colleagues also attempted to use natural products for AD treatment in a stereotaxic experimental rat model, including EPA-enriched ethanolamine plasmalogen (EPA-pPE) and EPA-enriched phosphatidylethanolamine (EPA-PE) from sea cucumbers. Both protocols increased neuronal plasticity and BDNF/TrkB/CREB signaling, with EPA-pPE showing a better preventive effect [171]. Choi and colleagues [172] investigated the neuroprotective properties of natural compounds for the treatment of Alzheimer’s disease (AD) in rats. They administered naringin, which is known for its antioxidant, anti-apoptotic, and anti-inflammatory properties, intrahippocampally to the rats that underwent stereotaxic induction of AD. The study found that naringin reversed the negative impact of Aβ accumulation on behavioral performance, reduced Bax levels, increased Bcl-2 levels, and restored the intrahippocampal levels of BDNF and TrkB. Furthermore, a recent study employed stereotaxic methodology for the application of intraventricular streptozotocin, resulting in pro-apoptotic effects and neuronal loss in the hippocampal regions. Tan IIA, the primary constituent of traditional Chinese medicine Danshen, showed anti-inflammatory, antioxidant, and anti-apoptotic properties, suggesting that the upregulation of the CREB-BDNF-TrkB signaling pathway in the hippocampus may attenuate underlying AD pathophysiological mechanisms [173], as shown in Table 2. Furthermore, various investigations of AD induction in rats have utilized alternative methodologies (Table 2), including the systemic administration of bisphenol A, which resulted in behavioral and biochemical changes indicative of AD, particularly pro-apoptotic effects. Treatment with syringic acid—a free radical scavenger and anti-inflammatory agent—counteracted these effects by activating the CREB/BDNF/TrkB and anti-apoptotic signaling pathways [174]. Another study utilized a d-galactose-ovariectomized rat model, which demonstrated cognitive impairment, increased apoptosis, and neuroinflammation. The neuroprotective agent diminazene aceturate could counteract these effects [175]. In another study, male rats were treated with d-galactose, which led to hippocampal Aβ1-42 level increases, oxidative damage, neuroinflammation markers, and apoptosis indicator proteins. In this model, L-theanine supplementation, which is known to have antioxidant activity, reduced Bax and caspase-3 activation and improved all AD-related characteristics [176].

Rodent models have been widely utilized to investigate Alzheimer’s disease (AD), with experiments conducted on mice confirming the prevalence of these models (Table 3). Chronic copper exposure decreased hippocampal BDNF and TrkB and increased apoptotic processes, resulting in a significant cognitive decline. These behavioral and neurochemical events could be addressed by modulating cuproptosis and CREB signaling [177]. The induction of AD in mice has typically been performed using stereotaxic surgery methodology. Lou and colleagues [178] administered Aβ1–42 for AD induction, resulting in alterations typical for AD with decreased BDNF and cognitive impairment. However, curcumin, a bioactive substance with anti-inflammatory and neuroprotective properties, upregulated neurogenesis via enhancing β-catenin and BDNF and decreasing caspase-3 activity through the PI3K/Akt pathway and improved cognitive functions. Senescence-accelerated SAMP8 mice were used as AD models to investigate the potential benefits of a polysaccharide fraction of Phellinus ribis (PRG) on the grounds of verified neurodegenerative manifestations in aged mice. PRG supplementation induced a decrease in apoptotic cells and the accumulation of Aβ-amyloid, while also resulting in the significant recovery of cognitive functions and BDNF levels and the Bcl-2/Bax ratio [179]. DL0410, an anti-AD agent known for its inhibition of AChE, reduced Aβ generation, the protection of mitochondrial respiration, and the inhibition of oxidative stress and neuroinflammation, confirming its neuroprotective capacity. It dose-dependently improved behavioral performance in AD mice, increased BDNF and TrkB expression, and alleviated neuronal apoptosis via the ERK1/2 and PI3K-AKT-GSK-3β pathways, and upregulated the Bcl-2/Bax ratio. The authors suggested that the NMDAR-CREB-BDNF pathway could create a positive feedback loop between neurotrophy and synaptic plasticity, with CREB at the core [180].

Furthermore, one study focused on the effects of genetic interventions on Alzheimer’s disease (AD) in mice. APP/PS1 double-transgenic C57BL/6 mice were used as an AD model. Behavioral testing confirmed significant cognitive impairment in transgenic mice, which correlated with hippocampal neuronal cell loss and injury, increased pro-apoptotic activity, and reduced expression of p-CREB and BDNF. Supplementation with 3,6′-disinapoyl sucrose (DISS) demonstrated a beneficial effect by shifting all evaluated AD indicators closer to control values [181]. Liu and colleagues [182] investigated the potential benefits of Qifu Yin, a plant component known to improve cognitive dysfunction and enhance learning and memory. In higher doses, Qifu Yin compensated for scopolamine-induced AD by improving cognitive functional behavior, decreasing oxidative and apoptotic indicators like JNK mRNA, and restoring hippocampal BDNF levels. Hwang and coworkers [183] employed fat-1 transgenic mice with a C57BL/6 background for the evaluation of scopolamine-induced amnesia. The higher endogenous omega-3 PUFA quantity prevented granular cell loss, increased BDNF signaling, and decreased apoptosis signaling with decreased caspase-3, Bax, and p53 expression in scopolamine-treated fat-1 mice. Additionally, in an AD experimental model of C57BL/6N mice, Ikram and colleagues [184] assessed the effects of cycloastragenol (20 mg/kg/day, orally for 6 weeks), as shown in Table 3. Cycloastragenol from Astragalus radix suppressed oxidative stress, BDNF, MAP kinases, and apoptosis markers like Bax and caspase-3 in the mice’s frontal cortex and hippocampus, preventing AD-related neurodegeneration. Anand and other researchers [185] induced an Alzheimer’s disease (AD) model in Swiss albino mice using aluminum chloride and d-galactose. They administered vanillin, an antioxidant compound, which improved oxidative stress in the hippocampal and cortical tissues, reduced caspase-3 levels, restored BDNF levels to near control values and enhanced cognitive function as shown in the novel object recognition test. In a separate study conducted by Broderick and colleagues [186], a triple-transgenic mouse model of AD underwent a five-month regimen that combined exercise training with resveratrol treatment. This therapeutic strategy not only increased BDNF levels but also resulted in lower apoptotic marker levels—including caspase-3, -7, and -9 and Adam-10—and a decrease in the buildup of harmful Aβ species.

Therapeutics that enhance the protease activity of tissue plasminogen activator, furin, plasmin, and MMPs, which convert proBDNF to mature BDNF, may aid in treating neurodegenerative disorders, including Alzheimer’s disease [126]. Gerenu et al. studied cognitive stimulation in AD using a Tg2576 mouse model, discovering that delayed memory decline correlated with increased proBDNF levels. They proposed that inadequate proteolytic processing might elevate proBDNF, leading to apoptosis and synaptic weakening. Furthermore, they demonstrated that upregulating plasminogen activator inhibitor-1 (PAI-1) via the JNK/c-Jun pathway inhibited the conversion of proBDNF to BDNF. The pharmacological inhibition of PAI-1 by PAI-039 reversed Aβ-induced tau hyperphosphorylation and neurotoxicity in aged mice, thereby enhancing cognitive function and BDNF maturation. The authors proposed BDNF maturation targeting as a potential treatment for AD [187]. Other research has found that chronic exposure to microcystin-LR for 180 days induces AD-like changes in six-week-old BALB/c mice, leading to the accumulation of Aβ and Tau-P in the brain and cognitive impairment. Neuronal cell apoptosis is observed predominantly in the CA1 and CA3 hippocampal regions, with an increased proBDNF/BDNF ratio and decreased TrkB and tPA expressions. Also, microcystin-LR induced p-JNK and C-Jun expression and inhibited p-CREB and p-PKC expression, promoting apoptosis [188].

Numerous studies have indicated the potential therapeutic effects of various compounds on memory impairment and neuroprotection in mouse models of AD. In this context, melatonin was discovered to be capable of reducing scopolamine-induced apoptosis and preventing mitochondrial damage by activating signaling from Akt/ERK/CREB and enhancing memory processes through antioxidant and anti-inflammatory properties in an AD mouse model [189]. Similarly, a combination of Ginkgo biloba with Hericium erinaceus extracts reduced memory deficit, inhibited apoptosis, and restored BDNF levels in a scopolamine-induced amnesia mouse model [190]. Additionally, Li and colleagues [191] established an AD mouse model by lateral ventricular injection with Aβ25-35. They examined the effect of a novel PPARα/γ dual agonist (N15), an anti-apoptotic, anti-inflammatory, and antioxidant agent, on AD mice. N15 suppressed Aβ-amyloid formation, enhanced BDNF mRNA expression, decreased presenilin-1 and β-secretase-1 expression, and enhanced Adam-10 mRNA expression. These actions prevented neuronal apoptosis, suggesting that N15 might be an AD drug candidate (Table 3).

In an earlier study, Banik and associates [192] induced AD in mice through Aβ42 injections and found that the transplantation of human umbilical cord blood-derived stem cells improved neuroprotection, as indicated by the downregulation of the apoptotic marker Fas-L and upregulation of CREB and BDNF. This therapy also enhanced spatial memory in the Morris water maze. Furthermore, Lin et al. [193] investigated the effects of electroacupuncture on cognitive impairment in an AD model involving APP/PS1 double-transgenic mice. They demonstrated that electroacupuncture at the Baihui acupoint, which is used in China for clinical therapy of cognitive impairment, prevented cognitive decline and neuronal apoptosis while restoring the balance of BDNF/proBDNF and TrkB/p75NTR in the hippocampus [194]. Moreover, a study on TAU-P301L transgenic zebrafish models of AD demonstrated that a significant decline in BDNF levels leads to neurodegenerative features such as axonal outgrowth shortening and neuronal cell death. The use of a TrkB antagonist, ANA-12, worsened these effects, whereas exogenous BDNF and therapy with a TrkB agonist, 7,8-dihydroxyflavone, improved axonal growth and behavioral performance [194]. These findings suggest that targeting the BDNF pathway may be a promising Alzheimer’s treatment (Table 2).

Numerous studies have investigated the impact of BDNF/pro-BDNF balance on apoptotic mechanism regulation in cell cultures designed to mimic AD (Table 4). Wang and colleagues [195] examined the effects of Aβ-induced cytotoxicity on primary neurons and astrocytes in neonatal Sprague Dawley rats and the neuroprotective effects of an α2-adrenoceptor agonist, dexmedetomidine. The administration of dexmedetomidine prevented β-amyloid toxicity by increasing BDNF production and BDNF mRNA expression, regulating histone deacetylase-2 and -5, and inhibiting apoptosis. The benefits of BDNF upregulation were confirmed through the use of recombinant BDNF and a selective inhibitor of histone deacetylases, Trichostatin A. Furthermore, Weng and coworkers [196] evaluated the potential of inhibiting Tau misfolding in human neuroblastoma SH-SY5Y cells for eight different agents. Some of the agents applied, such as ZN-015 (a coumarin derivative), VB-030, and VB-037 (quinoline derivatives), demonstrated significant neuroprotective effects, including a reduction in AD-like markers such as Bax and caspase-1/6, while also increasing the ratios of BDNF/proBDNF and TrkB/pTrkB. Catanesi and coworkers [197] also conducted a study on SH-SY5Y neuroblastoma cells, analyzing the efficacy of choline alfoscerate (α-GPC), a derivative of phosphatidylcholine. This compound enhanced cholinergic transmission and the activation of the BDNF/TrkB survival pathway, thereby suppressing the apoptotic effects of β-amyloid through the inhibition of caspase-9 activation.

The study highlights that α-GPC induces BDNF upregulation, which aligns with previous findings by Krishtal et al. (2017) regarding its neuroprotective effects in human neuroblastoma SH-SY5Y cells after treatment with exogenous BDNF and retinoic acid [198]. Furthermore, recent studies indicated the therapeutic potential of astragaloside IV (derived from Radix Astragali) and icaritin in the treatment of an in vitro model of Alzheimer’s disease (AD) in HT22 hippocampal cells through their modulation of brain-derived neurotrophic factor (BDNF) and apoptotic signaling. As shown in Table 4, astragaloside IV, a proliferator-activated receptor (PPARγ) agonist, was shown to reverse the toxic effects of amyloid β on BDNF and TrkB signaling, thereby reducing caspase-3 activation and neuronal apoptosis [199]. Similarly, icaritin, a prenylflavonoid derivative of Epimedium brevicornum Maxim, was effective in upregulating BDNF expression and the Bcl-2/Bax ratio despite the presence of Aβ25–35, ultimately reducing apoptosis [200]. Another study also indicated that cholesterol overloading disrupted BDNF/TrkB signaling and increased apoptosis in SH-SY5Y neuroblastoma cells, emphasizing the importance of such pathways in AD pathology and future treatments [201]. Moreover, Chen and colleagues examined how Farnesoid X Receptor (FXR) expression affected AD-related markers in SH-SY5Y cells. They found that Aβ-induced FXR overexpression led to increased neuronal apoptosis with an increase in the Bax/Bcl-2 ratio and suppression of the CREB/BDNF pathway [202]. Guo and colleagues [203] explored Alzheimer’s disease (AD) cell injury models using Aβ25-35 in PC12 cells, resulting in increased BDNF-AS and decreased BDNF, leading to increased apoptosis markers like Bax and caspase-3. Silencing BDNF-AS with long non-coding RNA increased BDNF levels and decreased apoptosis, suggesting that it could be used to treat AD-like models. In addition, Spielman and colleagues [204] found that the incretin hormones glucagon-like peptide-1 (GLP-1) and glucose-dependent insulinotropic polypeptide (GIP) inhibited apoptosis by reducing caspase-3/7 activation and also increased BDNF levels in THP-1 and BV-2 murine microglial cells. In a recent study, Pinky and colleagues [205] evaluated the role of paeoniflorin, a pyruvate dehydrogenase kinase 3 antagonist, in the neurodegenerative cascade involving scopolamine-treated N2A mouse neuroblasts. They showed that paeoniflorin enhanced BDNF activity by modulating neuronal function and reducing apoptosis progression, potentially serving as a treatment for AD. Further, Tagai and colleagues [206] found that increased BDNF levels prevent AD-like features and inhibited caspase-6 activation in neuron-like differentiated human neuroblastoma SH-SY5Y cells. Additionally, a group of researchers examined the therapeutic efficacy of lycopene, a potent β-carotenoid antioxidant, in relation to neurodegenerative disorders. Lycopene increased the Bcl-2/Bax ratio and decreased caspase-3 activation in mouse neural stem cells to restore neurodegenerative markers and prevent tert-butyl hydroperoxide-induced apoptosis [207]. Similarly, Zhang and colleagues [208] used shrimp Pandalus borealis extract, which contains 16.84% of marine unsaturated fatty acids, to treat Aβ-induced neurodegeneration. The extract demonstrated a significant anti-apoptotic effect, resulting in a decrease in the Bax/Bcl-2 ratio and caspase-3, as well as an increase in cell viability. However, it had no effect on the levels of BDNF and TrkB (Table 4). Similarly, Wen and coworkers [209] found that tert-butyl hydroperoxide treatment resulted in AD-like neuronal damage, but secretion from bone marrow mesenchymal stem cells, pretreated with berberine, prevented these neurodegenerative features by increasing BDNF and Bcl-2 levels and reducing Bax and caspase-3 activity. Apart from this, studies utilizing samples from human patients with AD and cell cultures from 5xFAD male mice have highlighted the varying effects of BDNF treatment and cerebrospinal fluid derived from patients with AD on neurodegenerative processes. While Fleitas and colleagues [210] reported that cerebrospinal fluid from patients with AD raised apoptosis in hippocampal neural stem cells, probably as a result of a notable rise in proBDNF, p75NTR, and sortillin levels, Song and colleagues [211] showed that BDNF from mesenchymal stem cells could prevent death and improve cell survival in AD-like cells. These results highlight the multifaceted function of BDNF and its metabolites in the pathophysiology of Alzheimer’s disease.

**Table 4:** The implications of the BDNF/proBDNF relationship in apoptotic-related AD-like disorders in in vitro models.

	BDNF/proBDNF	Neurotrophin Receptor	Apoptotic Mechanism	Treatment	Effect
[195] (hippocampal and cortical rat cell lines)	BDNF ↓	-	Apoptotic rate ↑	Dexmedetomidine (specific agonist of α2-adrenoceptor)	BDNF ↑ Apoptotic rate ↓
[196] (human neuroblastoma SH-SY5Y cells)	BDNF ↓ proBDNF ↑	pTrkB ↑ TrkB ↓	Bax ↑ Bcl-2 ↓ Caspase-1 and -6 ↑ Neuronal growth ↓	ZN-015, VB-030, and VB-037 (anti-inflammatory, antioxidant, and antimicrobial)	BDNF/proBDNF ↑ TrkB/pTrkB ↑ Bax ↓ Bcl-2 ↑ Caspase-1 and -6 ↓ Neuronal growth ↑
[197] (human neuroblastoma SH-SY5Y cells)	BDNF ↓	pTrkB ↑	Pro-caspase-9 ↓ Caspase-9 ↑ Neurite length ↓	α-GPC (cholinergic transmission stimulator)	BDNF ↑ pTrkB ↓ Pro-caspase-9 ↑ Caspase-9 ↓ Neurite length ↑
[198] (human neuroblastoma SH-SY5Y cells)	BDNF ↓	-	Caspase-3/7 ↑ Neurite length ↓	Retinoic acid plus exogenous BDNF	BDNF ↑ Caspase-3/7 ↓ Neurite length ↑
[199] (mouse hippocampal HT22 cell line)	BDNF ↓	pTrkB ↑ TrkB ↓	Caspase-3 ↑ Neuronal cell death ↑	Astragaloside IV (PPARγ agonist)	BDNF ↑ TrkB/pTrkB ↑ Caspase-3 ↓ Neuronal cell death ↓
[200] (mouse hippocampal HT22 cell line)	BDNF ↓	-	Bax ↑ Bcl-2 ↓ Caspase-3 ↑ Apoptotic rate ↑	Icaritin (anti-inflammatory)	BDNF ↑ Bax ↓ Bcl-2 ↑ Caspase-3 ↓ Apoptotic rate ↓
[202] (SH-SY5Y cells and mouse hippocampal neurons)	BDNF ↓	-	Bax ↑ Bcl-2 ↓ Apoptotic cells ↑	-	BDNF ↑ Bax ↓ Bcl-2 ↑ Apoptotic cells ↓
[203] (PC12 cells—rat pheochromocytoma cell line)	BDNF ↓	-	Bax ↑ Caspase-3 ↑ Bcl-2 ↓ Cell viability ↓	Long non-coding RNA BDNF-AS	BDNF ↑ Bax ↓ Caspase-3 ↓ Bcl-2 ↑ Cell viability ↑
[204] (THP-1 and BV-2 cells)	BDNF ↓	-	Caspase-3/7 ↑	GLP-1 GIP	BDNF ↑ Caspase-3/7 ↓
[205] (N2A cells—mouse neuroblasts)	BDNF ↓	-	Apoptotic cells ↑	Paeoniflorin (PDK3 blocker)	BDNF ↑ Apoptotic cells ↓
[206] (ndSH-SY5Y cells)	BDNF ↓	-	Neurite length ↓ Cell viability ↓ Neurite fragmentation ↑	Exogenous BDNF	BDNF ↑ Neurite length ↑ Cell viability ↑ Neurite fragmentation ↓
[207] (mouse neural stem cells)	BDNF ↓	-	Bax ↑ Caspase-3 ↑ Bcl-2 ↓ Apoptotic cells ↑	Lycopene (antioxidant)	BDNF ↑ Bax ↓ Caspase-3 ↓ Bcl-2 ↑ Apoptotic cells ↓
[208] (SH-SY5Y cells)	BDNF ↓	TrkB ↓	Bax ↑ Caspase-3 ↑ Bcl-2 ↓ Cell viability ↓	*Pandalus borealis* extract	BDNF n.c. TrkB n.c. Bax ↓ Caspase-3 ↓ Bcl-2 ↑ Cell viability ↑
[209] (mouse cortical neurons)	BDNF ↓	-	Bax/Bcl-2 ratio ↑ Cleaved caspase-3/caspase-3 ratio ↑ Cell viability ↓	Berberine (anti-inflammatory, antioxidant, Ach-esterase inhibitor)	BDNF ↑ Bax/Bcl-2 ratio ↓ Cleaved caspase-3/caspase-3 ratio ↓ Cell viability ↑
[210] (hippocampal neural stem cells from APP/PS1 transgenic mice)	proBDNF ↑	p75 ↑ sortilin ↑	Apoptotic cells ↑	CSF of patients with AD	proBDNF ↑↑ p75 ↑↑ Sortilin ↑↑ Apoptotic cells ↑↑
[211] (5xFAD mouse neuronal cells)	BDNF ↓	pTrkB ↓	Cell viability ↓ Nuclear apoptosis ↑ Cleaved caspase-3 ↑	Exogenous BDNF	BDNF ↑ Cell viability ↑ Nuclear apoptosis ↓ Cleaved caspase-3 ↓

### 4.2. Role of BDNF/proBDNF in Apoptotic Mechanisms Involved in Parkinson’s Disease

Parkinson’s disease (PD) is the second-most common age-related neurodegenerative disorder. Recent studies on rodent models of Parkinson’s disease (PD) have improved our understanding of its mechanisms and treatments (Table 5). Consistent with this, one study used 6-hydroxydopamine, a surgical induction of Parkinson’s disease (PD), in rats, which resulted in motor deficits and an increase in the apoptosis of dopaminergic neurons. This was evidenced by parameters such as an increase in the Bax/Bcl-2 ratio and p38, along with a decline in the activation of Akt [212]. In addition, the transplantation of BDNF-modified human umbilical cord mesenchymal stem cell-derived dopaminergic-like neurons in 6-OHDA-induced PD in rats has shown promise in enhancing motor function as well as increasing levels of dopamine, BDNF, and TrkB. However, there are concerns regarding the limited effects of BDNF and its capacity to cross the blood–brain barrier [213]. These findings underscore the need for further research to maximize therapeutic strategies for PD.

**Table 5:** The implications of the BDNF/proBDNF relationship in apoptotic-related PD-like disorders in in vivo PD models.

	BDNF/proBDNF	Neurotrophin Receptor	Apoptotic Mechanism	Treatment	Effect
[212] (rat stereotaxic 6-OHDA model)	-	-	Bax/Bcl-2 ratio ↑ P38 ↑ Akt ↓ Number of striatal DA neurons ↓	BHME (BDNF mimetic, antioxidant)	Bax/Bcl-2 ratio ↓ P38 ↓ Akt ↑ Number of striatal DA neurons ↑
[213] (rat stereotaxic 6-OHDA model)	BDNF ↓	TrkB ↓	Number of SN and ST DA neurons ↓	Exogenous BDNF (umbilical stem cells)	BDNF ↑ TrkB ↑ Number of SN and ST DA neurons ↑
[214] (rat stereotaxic rotenone model)	BDNF ↓	-	Number of striatal DA neurons ↓	Apigenin (antioxidant, anti-apoptotic, anti-inflammatory)	BDNF ↑ Number of striatal DA neurons ↑
[215] (rat rotenone systemic administration model)	BDNF ↓	TrkB ↓	Caspase-3 ↑ Striatal neuron degeneration ↓	Roflumilast (PDE4 inhibitor)	BDNF ↑ TrkB ↑ Caspase-3 ↓ Striatal neuron degeneration ↑
[216] (rat stereotaxic rotenone model)	BDNF ↓	-	Number of striatal neurons ↓	Agmatine (antioxidant, anti-inflammatory, anticonvulsant, antidepressant)	BDNF ↑ Number of striatal neurons ↑
[217] (mouse rotenone systemic administration model)	BDNF ↓	pTrkB ↓	Bax ↑ Bcl-2 ↓ Caspase-3 ↑ Loss of SN and ST DA neurons ↑	Oleuropein (antioxidant)	BDNF ↑ pTrkB ↑ Bax ↓ Bcl-2 ↑ Caspase-3 ↓ Loss of SN and ST DA neurons ↓
[218] (mouse MPTP model and MPP+ rat model)	BDNF ↓	-	Bcl-2 ↓ Number of striatal neurons ↓	Electroacupuncture	BDNF ↑ Bcl-2 ↑ Number of striatal neurons ↑
[219] (mouse MPTP model)	BDNF n.c.	TrkB n.c.	TH-positive neurons in SN ↓ Apoptotic cells in SN ↑	K252a (TrkB antagonist) NaHS (H_2_S donor)	↑TH-positive neurons in SN Apoptotic cells in SN ↓
[220] (mouse MPTP model)	BDNF ↓	-	Bax ↑ Bcl-2 ↓ Number of striatal neurons ↓	DA-JC1 (GLP-1 and GIP agonist)	BDNF ↑ Bax ↓ Bcl-2 ↑ Number of striatal neurons ↑
[221] (mouse MPTP model)	BDNF ↓	-	Bax ↑ Bcl-2 ↓ Caspase-3 ↑ TH-positive neurons in SN ↓	Wuzi Yanzong pill (antioxidant, anti-apoptotic)	BDNF ↑ Bax ↓ Bcl-2 ↑ Caspase-3 ↓ TH-positive neurons in SN ↑
[222] (suppression of NA stimulatory effect)	BDNF ↓	pTrkB ↓ TrkB ↓	Neuronal survival ↓	BDNF stimulation from LC	BDNF ↑ pTrkB ↑ TrkB ↑ Neuronal survival ↑
[223] (mouse stereotaxic 6-OHDA model)	BDNF ↓	-	Caspase-3 ↑ Caspase-9 ↑ Striatal degeneration and gliosis ↑	*Schisandra chinensis* extract (antioxidant, antidepressant)	BDNF ↑ Caspase-3 ↓ Caspase-9 ↓ Striatal degeneration and gliosis ↓
[224] (MPTP mouse model and MPP+ SH-SY5Y cell model)	BDNF ↓	-	DA cell apoptotic rate ↑	miR-125b-5p	BDNF ↑ DA cell apoptotic rate ↓

Various studies have employed a stereotaxic model to induce Parkinson’s disease (PD) in rats using rotenone, which has highlighted the neuroprotective effects of various therapeutic agents (Table 5). Anusha et al. (2017) demonstrated that rotenone induced a PD-like disorder, which was characterized by behavioral alteration and decreased BDNF, striatal dopaminergic number of neurons, and dopamine D2 receptor expression [214]. Apigenin, which possesses anti-apoptotic, anti-inflammatory, and antioxidative properties, demonstrated a significant increase in BDNF levels and a remarkable improvement in behavioral and biochemical parameters when compared to rotenone-treated rats [214]. Farid and colleagues [215] similarly demonstrated the neurotoxic effects of rotenone in rats, resulting in decreased BNDF and TrkB levels and elevated caspase-3. They conducted neuroprotective experiments with a control (L-DOPA) and roflumilast (0.2, 0.4, or 0.8 mg/kg, per os) simultaneously with the rotenone protocol for 21 days. Roflumilast, a PDE4 inhibitor, enhanced motor activity and coordination, restored BDNF/TrkB/CREB and PI3K/Akt survival signaling cascades, and preserved dopaminergic neuron structure and architecture in the striatum. Bilge and colleagues [216] applied a stereotaxic PD model with rotenone in rats to estimate the neuroprotective action of agmatine, an endogenous decarboxylation product of l-arginine, which was found to mitigate motor impairments, decrease striatal neuronal death, and restore the BDNF/CREB survival pathway. On the other hand, Singh and coworkers [217] used rotenone to induce experimental PD in a mouse model. The protocol resulted in motor impairment, a decrease in p-TrkB and BDNF levels, and increased apoptosis, characterized by elevated Bax and caspase-3 levels, along with a reduction in Bcl-2. To counteract the neurotoxic effects of rotenone, antioxidant supplementation with oleuropein, a phenolic compound, was administered in various doses. Higher doses of oleuropein demonstrated a neuroprotective effect against rotenone-induced Parkinson’s disease (PD), effectively restoring the BDNF/CREB/Akt pathway, reducing apoptotic markers, and improving motor performance (Table 5).

On the grounds of previously established rodent PD models, namely, MPTP (1-methyl-4-phenyl-1,2,3,6-tetrahydropyridine hydrochloride) in mice and an MPP+-lesioned rat model (1-methyl-4-phenylpyridinium), Lin and coworkers [218] explored the therapeutic effects and mechanisms of electroacupuncture at the GB34 (Yanglingquan) and LR3 (Taichong) acupoints according to Chinese traditional medicine. As presented in Table 5, both PD models resulted in characteristic alterations: behavioral (motor impairment: rotarod test in mice and apomorphine-induced rotation behavior and locomotor activity in rats), biochemical (decline in BDNF, dopamine content, and Bcl-2), and histological (decrease in the number of striatal dopaminergic neurons). Electroacupuncture protocols succeeded in diminishing motor symptoms of PD in both rodent models. An interesting model for PD induction in mice was presented by Hacioglu and collaborators [219]. The authors intraperitoneally administered 1-methyl-phenyl-1,2,3,6-tetrahydropyridine (MPTP neurotoxin) alone and along with a TrkB antagonist—K252a—which resulted in clear PD-like outcomes using behavioral (balance beam test and pole test) and histological (the number of TH-positive neurons and apoptotic cells in the substantia nigra) criteria, but not with biochemical markers (BDNF and TrkB). In addition, potential neuroprotection was evaluated by NaHS (H2S donor) administration, which sufficiently recovered all estimated parameters. The analysis of the obtained results supports the conclusion that the use of TrkB receptor agonists in combination with H2S may be promising for the treatment of PD. Another investigation based on the MPTP-induced PD mouse model was previously carried out by Ji and other researchers [220] to investigate a novel dual GLP-1 and GIP receptor agonist (DA-JC1)’s neuroprotective role. In accordance with this PD model’s reliability, the applied protocol fulfills the criteria for PD confirmation. The administration of DA-JC1 reduced or reversed most of the MPTP-induced motor impairments in the rotarod and in a muscle strength test. At the same time, this agonist increased the Bcl-2/Bax ratio, recovered BDNF levels, and restored tyrosine hydroxylase (TH)-positive neurons in the substantia nigra. Altogether, the obtained results support the potential therapeutic usage of this agent. The Wuzi Yanzong pill, an herbal seed complex that also contains Schisandra chinensis, traditionally used in Chinese alternative medicine (to treat male infertility and kidney deficiency), was applied by Hang and colleagues [221] on the grounds of MPTP-induced PD. The authors reported that Wuzi Yanzong pill administration improved motor function in the MPTP-induced PD model, prevented the loss of TH-positive neurons in the substantia nigra, and lowered the expression of Bax and cleaved caspase-3, but increased the expression of Bcl-2 and BDNF. These neuroprotective effects may advocate the use of this herbal complex as an adjuvant therapeutic approach for the treatment of PD features.

Moreover, Hassani et al. performed an elaborate experimental design in mice to examine the interaction between dopaminergic midbrain neurons, which are impaired in PD, and locus coeruleus neurons, the primary source of noradrenergic projections [222]. They identified that BDNF is a key mediator in the interaction with a positive effect on dopaminergic neurons and the modulation of the survival actions of locus coeruleus inputs achieved by amplifying BDNF/TrkB signaling and suppressing apoptosis [222]. Their findings, supported by evidence in transgenic mice, suggest that noradrenergic “preconditioning” might be capable of protecting dopaminergic neurons. Furthermore, Yan and collaborators employed the stereotaxic 6-OHDA-induced PD model in mice to test neuroprotection from Schisandra chinensis, a traditional Chinese herb [223]. This antioxidant improved motor performance and increased BDNF expression while reducing apoptotic markers such as caspase-9/3 and p53. The authors proposed that neuroprotection may be mediated through the BDNF/Nrf2/NF-κB pathway.

To further utilize in vivo and in vitro PD models, allowing complex insight into this intriguing topic, Fan and coworkers [224] established an MPTP-induced mouse model of PD along with an MPP+-induced SH-SY5Y cell model for the evaluation of long non-coding RNA BDNF-AS’s impact on autophagy and apoptosis. According to the presented results, PD induction protocols resulted in BDNF-AS upregulation (in a dose-dependent manner), while miR-125b-5p was downregulated. On the other hand, BDNF-AS knockdown was found to significantly promote cell proliferation, accompanied by apoptosis and autophagy inhibition. The authors proposed that silencing BDNF-AS induced an increase in the number of TH-positive neurons. Also, they claimed that miR-125b-5p was involved in the effects of BDNF-AS on SH-SY5Y cell apoptosis and autophagy.

Finally, it is important to note that among the primary rodent models of Parkinson’s disease, Nadig and coworkers also developed a zebrafish model of Parkinson’s disease (PD) induced by MnCl_2_, which exhibited both motor and non-motor symptoms of PD, along with oxidative stress, neuroinflammation, and neuronal apoptosis in the brain, demonstrating its potential as a cost-effective drug discovery model [225].

In recent in vitro studies involving MPP+-induced PD model experiments, significant results were obtained regarding neuroprotective agents (Table 6). Yu et al. examined the effect of 2,3,5,4′-tetrahydroxystilbene-2-O-β-D-glucoside (THSG), a compound extracted from Polygonum multiflorum, on the ability to mitigate cytotoxicity caused by the Parkinsonian toxin MPP+ in rat dopamine neurons and SH-SY5Y human neuroblastoma cells [226]. They discovered that THSG protected neurons via the BDNF-TrkB and FGF2-Akt signaling pathways by increasing Bcl-2 levels and decreasing caspase-3 activation. In MPTP-treated C57BL/6J mice, THSG improved dopaminergic neuronal integrity and reduced clinical symptoms in vivo [226]. In addition, Deng et al. examined the mechanisms of pramipexole (PPX), a common PD drug, in human neuroblastoma cell lines [227]. They found that PPX inhibited miR-494-3p, which promotes PD progression, and increased BDNF levels. The study suggested that miR-494-3p and BDNF modulation by PPX reduced MPP+-induced neurotoxicity through anti-apoptotic, anti-inflammatory, and antioxidative effects. Overall, the findings from these studies indicate potential therapeutic strategies for PD treatment [227].

In another study, Zhu et al. (2019) [228] investigated norepinephrine’s neuroprotective effect on dopaminergic neurons in rat ventral mesencephalon primary culture and MN9D dopaminergic cells. Norepinephrine was found to inhibit 6-hydroxydopamine-induced apoptosis and increase tyrosine hydroxylase expression in a time- and dose-dependent manner. This effect is correlated with an increased level of BDNF and phosphorylated ERK1/2 in treated cultures. Transfecting cells with BDNF cDNA or peptides produced the same anti-apoptotic effects [228].

Moreover, Ceccarini et al. (2022) [229] examined the neuroprotective effects of ω-3 polyunsaturated fatty acids (PUFAs), eicosapentaenoic acid (EPA), and docosahexaenoic acid (DHA) on BDNF and GDNF expression in SH-SY5Y neuroblastoma cells. They showed that EPA inhibited the neurotoxic activity of 6-hydroxydopamine, restored mitochondrial function, and stimulated BDNF and GDNF expression through epigenetic mechanisms, but DHA was unable to exhibit such effects [229]. Furthermore, Cheon and colleagues [230] investigated heat-killed Lactobacillus plantarum 200655, which revealed its neuroprotective effect against H_2_O_2_-treated SH-SY5Y cells by increasing the levels of BDNF and tyrosine hydroxylase and preventing apoptosis by enhancing the ratio of Bcl-2/Bax and decreasing caspase-3, which suggested that probiotics may prevent neurodegenerative diseases. In addition, Chen et al. (2020) studied hydroxy-4′-methoxychalcone (AN07), exhibiting its cytoprotective and antioxidant effects against LPS-induced inflammation and methylglyoxal-induced neurotoxicity in SH-SY5Y cells, promoting cell survival and neurotrophic BDNF expression while inhibiting cytochrome c release and blocking mitochondrial apoptosis [231]. These findings underscore the potential of drugs that target BDNF-related apoptotic pathways in developing therapeutic strategies for neurodegenerative disorders such as Parkinson’s disease.

### 4.3. Role of BDNF/proBDNF in Apoptotic Mechanisms Involved in Huntington’s Disease

Recent investigations utilizing mouse models of Huntington’s disease (HD) have enhanced our comprehension of its processes and therapeutic approaches (Table 7). Accordingly, Li et al. proposed a new experimental model of HD by using heterozygous and pro-BDNF knockout mice, with the latter exhibiting phenomenal weight loss, declined reflexes, abnormal motor activities, and reduced lifespan, demonstrating an HD-like phenotype [232]. The experiment examined several features of behavior, suggesting that heterozygous mice exhibited impaired learning, memory deficits, and depression-like behavior compared to wild-type mice. Neurochemical research revealed reduced levels of BDNF in cortical and cerebellar samples, with no significant alterations in TrkB, p75NTR, and sortillin receptors. Elevated levels of caspase-3 and reduced levels of Bcl-2 indicated enhanced pro-apoptotic pathways, alongside reduced cell density and increased nuclear pyknosis in the dentate gyrus, implying the model’s relevance to Huntington’s disease [232]. In contrast, Wehner et al. studied an intervention with the p75 neurotrophin receptor in a Q175 knock-in mouse model, observing no significant reduction in neurotrophin receptor expression yet a significant decrease in anti-apoptotic mechanisms such as Bcl-Xl and XIAP expression, as well as striatal volume, over time, without conducting behavioral assessments [233]. Researchers have explored various interventions in genetic models of Huntington’s disease (HD) using R6/2 mutant mice, which mimic human HD symptoms (Table 7). In line with this, Cardinale and colleagues [234] found that administering the PARP-1 inhibitor INO-1001 improved behavioral performance and neuroprotection by increasing BDNF and pCREB expression and reducing the Bax/Bcl-2 ratio, thereby inhibiting apoptosis. Simultaneously, Smail’s team employed olfactory bulb neuroblasts to boost BDNF activity, which reduced apoptosis but did not completely restore granule cell survival [235]. Moreover, Solés-Tarrés and colleagues [236] utilized PACAP (pituitary adenylate cyclase-activating polypeptide) in the R6/1 mouse model of Huntington’s disease, which exhibited anti-apoptotic and neurotrophic benefits, resulting in enhanced motor functions and reduced caspase-3 activity. Their findings indicate that targeting PAC1 receptors may provide therapeutic potential for the treatment of HD.

In addition, Ahmed and associates [237] investigated the neuroprotective properties of 7,8-dihydroxyflavone (7,8-DHF), a TrkB agonist, using in vitro and in vivo models of neuronal death induced by 3-nitropropionic acid (3-NP). Their findings demonstrated that 3-NP injection resulted in motor deficits and reduced levels of BDNF, pTrkB/TrkB, and pCREB/CREB, as well as elevated caspase-3 and apoptotic cells in the striatum. Treatment with 7,8-DHF mitigated these neurotoxic effects, indicating possible therapeutic advantages for Huntington’s disease (HD). Similarly to this, Abdelfattah’s study employed the antioxidants rutin and selenium, which improved oxidative balance, restored the anti-apoptotic marker Bcl-2, decreased pro-apoptotic Bax and caspase-3, and enhanced BDNF levels [238]. Furthermore, research conducted by Ibrahim and Rasheed on rats validated the locomotor dysfunction and neurotoxic effects of 3-NP; however, treatment with diapocynin alleviated these symptoms by elevating BDNF and Bcl-2 levels while reducing p53 and Bax levels [239]. In addition, Gendy’s research emphasized berberine’s protective function, improving behavioral performance and anti-apoptotic indicators, including a reduction in caspase-3 and a rise in Bcl-2, BDNF, and the pTrkB/TrkB ratio, as well as the striatal lesion score [240]. Finally, Kaur’s research on SH-SY5Y cells revealed that 4-(methylthio) butyl isothiocyanate (4-MTBITC) mitigated neurotoxicity induced by 3-NP by decreasing apoptosis through caspase-3 suppression and elevating BDNF and TrkB levels [241]. These studies highlight the efficacy of various neuroprotective drugs in mitigating the neurotoxic effects generated by 3-NP in Huntington’s disease mice.

## 5. Emerging Therapeutic Strategies for Neurodegenerative Diseases: Targeting BDNF and proBDNF Apoptotic Signaling

**Figure 3:**
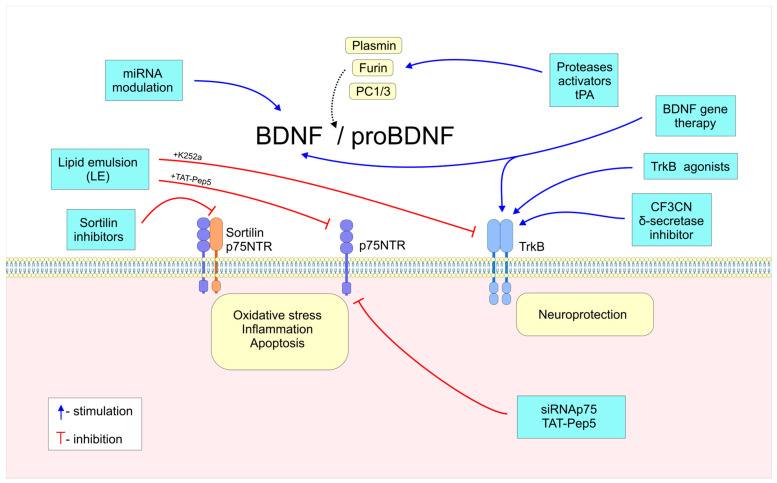
Current therapeutic strategies targeting BDNF and proBDNF signaling scheme.

As is evident from the presented data, the conventional and most commonly used treatments for neurodegenerative diseases such as Alzheimer’s, Parkinson’s, and Huntington’s focus on modifying various effector cascades that lead to characteristic symptomatology. Therefore, it is not surprising that protocols (Figure 3) based on anti-apoptotic, antioxidative, and anti-inflammatory treatments are gaining importance as therapeutic strategies [242,243]. However, therapeutic strategies aimed at modulating the balance between BDNF and proBDNF, as well as their receptors, while not as frequently utilized in experimental protocols, hold significant scientific importance due to their potential to target neurodegenerative processes. In general, these innovative therapeutic methodologies are based on two distinct strategies. Specifically, efforts are being made to elevate BDNF levels by either stimulating de novo synthesis in situ or administering exogenous BDNF from various sources to targeted brain regions associated with neurodegenerative pathology. Recent preclinical studies demonstrated a novel targeted therapeutic strategy for genetically at-risk patients using a wild-type AAV virus as a vector to overexpress BDNF in brain-injured BDNF-Val66Met mouse models. This intervention improved cellular, motor, and cognitive behavior outcomes; increased BDNF and TrkB levels; and decreased caspase-3-mediated apoptosis and the proBDNF/BDNF ratio [244]. A phase 1 clinical trial has started to evaluate the safety and efficacy of engineered AAV2-BDNF in patients with AD and those with mild cognitive impairment. The trial has reached the stage of recruiting patients [245]. Despite the potential of AAV-based gene therapies, there are significant concerns about their invasiveness, inflammatory responses, difficulties in precise gene expression control, and potential negative effects from prolonged BDNF expression, such as p75NTR activation and TrkB reduction [246].

Moreover, to achieve beneficial therapeutic effects, other studies have focused on improving the BDNF/proBDNF ratio through enhanced proteolytic activity. Long et al.’s recent study (2025) showed that different concentrations of IL-1β have a direct impact on neuronal apoptosis and survival. This regulation is linked to the regulation of the BDNF/proBDNF ratio by the conversion proteases furin and proprotein convertase 1/3 (PC1/3). Low IL-1β levels resulted in an increased BDNF/proBDNF ratio and elevated levels of furin and PC1/3, which inhibited neuronal apoptosis and triggered neuroprotection in mouse hippocampal neuronal cells. Conversely, elevated levels of IL-1β triggered apoptosis, lowered the BDNF/proBDNF ratio, and diminished the levels of furin and PC1/3, ultimately resulting in neurodegeneration [247]. An earlier study discovered that proBDNF signaling via the p75NTR receptor and reduced conversion of proBDNF to BDNF led to isoflurane anesthetic neurotoxicity. Blocking p75NTR signaling chemically or genetically with siRNAp75, TAT-Pep5, or plasmin and tPA activated the PI3K/Akt survival pathway and inhibited caspase-3-mediated apoptosis, reducing cytotoxicity in primary neurons and mouse pups. This finding suggested that preventing neurotoxicity caused by anesthetics depends on the conversion of proBDNF to BDNF. Neurotoxicity may arise from the downregulation of BDNF-TrkB signaling or the upregulation of proBDNF/p75NTR, yet the underlying mechanism remains unclear [248].

In accordance with this, some researchers have proposed a therapeutic approach that includes various interventions involved in BDNF/proBDNF signaling at the TrkB level (using agonists) and at the p75NTR level (using antagonists). One study indicated that the second-generation pharmacologically improved BDNF mimetic, the prodrug 7,8-DHF (R13), demonstrated significant therapeutic potential for AD. This benefit is achieved by activating TrkB signaling, preventing amyloid-beta (Aβ) deposition, activating the PI3K/Akt and MAPK/Erk survival pathways, and inhibiting pathological processes associated with AD. R13 has also been shown to effectively protect hippocampal synapses and enhance memory deficits, suggesting its potential as a treatment for AD [249]. Currently, R13 is undergoing a phase 1 clinical trial as a potential treatment for AD, with the results yet to be determined [250]. Additionally, research has examined the therapeutic efficacy of the BDNF/TrkB agonist, CF3CN, and the δ-secretase inhibitor, #11A, in human SNCA transgenic PD animal models. The results showed that the therapies enhanced motor function and dopaminergic neuron survival and inhibited neuronal apoptosis, with the best PD treatment being a combination treatment [251]. Jia et al. investigated the effects of lipid emulsion (LE) both alone and in combination with K252a, a TrkB inhibitor, or TAT-Pep5, a p75NTR inhibitor, on CNS toxicity induced by bupivacaine anesthetic (BPV) in rats. LE decreased BDNF expression, enhanced the survival of hippocampal neurons, and reduced apoptosis by increasing TrkB expression while not significantly impacting pro-BDNF/p75NTR levels. The addition of K252a to LE therapy resulted in increased expressions of p75NTR and BDNF, while proBDNF levels remained unchanged. The optimal synergy of LE and TAT-Pep5 in suppressing caspase-3-mediated apoptosis was achieved by downregulating TrkB and BDNF. The anti-apoptotic effects of these LE treatments are closely linked to their modulation of the proBDNF-p75NTR/BDNF-TrkB pathways [252].

Nonetheless, these therapeutic agents frequently encounter significant translational obstacles, such as short half-lives, insufficient permeability across the blood–brain barrier, and unexpected off-target effects. Obviously, to overcome these limitations, it is necessary to perform additional preclinical investigations to evaluate the possible feasibility of such agents for clinical application. Furthermore, therapies based on the antagonism of p75NTR or sortilin must be thoroughly assessed for potential developmental toxicity, considering the receptor’s involvement in axonal pruning and plasticity (Table 8). Consequently, future therapeutic strategies are expected to involve combinatorial approaches that deal with both neurotrophin processing (such as protease regulation) and receptor responsiveness, ideally customized to the disease stage and specific vulnerabilities of cell types.

## 6. Conclusions

The delicate equilibrium between BDNF and proBDNF is critical in regulating neuronal apoptosis in neurodegenerative diseases. While BDNF acts to mediate neuronal survival through TrkB, proBDNF, through p75NTR, can induce apoptotic cascades. The dysregulation of this fine balance, usually shifting in favor of proBDNF, results in neuronal degeneration in Alzheimer’s disease, Parkinson’s disease, and Huntington’s disease. Understanding the complex role of neurotrophins in brain diseases and health requires an understanding of the BDNF/proBDNF balance which regulates neuronal apoptosis. Our review presents the first integrated framework linking BDNF/proBDNF signaling with downstream apoptotic regulation and their regulatory mechanisms, like receptor dynamics and proteolytic and transcriptional regulation, across multiple neurodegenerative diseases, including AD, PD, and HD. Furthermore, we evaluate the practical applicability of therapeutically modifying this axis in humans, emphasizing both possible advancements and existing limitations. This systems-level approach provides a novel foundation for developing targeted, neurotrophin-based therapies for neurodegenerative diseases to slow the progression of disease and enhance quality of life for those who suffer from these devastating disorders.

## 7. Future Directions: Translational Application of BDNF/proBDNF Research

Future studies need to aim to translate scientific knowledge about BDNF and proBDNF interplay into practical strategies for application in clinical settings. One promising strategy is to use the ratio of BDNF/proBDNF as a diagnostic, prognostic, and follow-up therapeutic biomarker in neurodegenerative disorders. Changes in the BDNF/proBDNF ratio have been measured in the peripheral blood and cerebrospinal fluid of both animal models and human patients with Alzheimer’s, Parkinson’s, and Huntington’s disease. This finding suggests the possibility of developing non-invasive tests for early diagnosis, the monitoring of the illness, and the assessment of responses to treatment. However, major challenges persist, such as diversity in sample sources, a lack of consistent measurement methodologies, and low longitudinal validity. Furthermore, treatment methods designed to modify this ratio, such as TrkB receptor agonists, p75NTR receptor antagonists, protease modulators, or gene-regulatory strategies, need to be carefully tested in laboratories and clinical trials. Future research should aim to identify specific targets in BDNF/proBDNF signaling pathways that control neuron survival and cell death, which can be improved for treatment purposes. This effort should include a strong emphasis on personalized medicine strategies that consider the unique genetic and environmental factors contributing to each individual’s disease. Future research should also explore the potential for new combination therapies that merge both neurotrophin and non-neurotrophin therapeutic strategies to enhance the management of neurodegenerative disorders. Aside from curative methods, future investigations may enhance the application of knowledge, considering the BDNF/proBDNF ratio as a potential early diagnostic sign as well as a tool for following up on the progression of disease and therapy efficiency in neurodegenerative disorders.

## Figures and Tables

**Table 2:** The implications of the BDNF/proBDNF relationship in apoptotic-related AD-like disorders in experimental rat models.

	BDNF/proBDNF	Neurotrophin Receptor	Apoptotic Mechanism	Treatment	Effect
[158]	BDNF ↓	TrkB ↓	Bax ↑ Bcl-2 ↓ Caspase-3 ↑	*Angelica sinensis* polysaccharide (antioxidant)	BDNF/TrkB/CREB pathway ↑ Bax ↓ Bcl-2 ↑ Caspase-3 ↓
[159]	BDNF ↓	-	Bax ↑ Bcl-2 ↓ Caspase-3 ↑	Brain-specific angiogenesis inhibitor-1	STAT3/EZH2 pathway ↑ BDNF ↑ Bax ↓ Bcl-2 ↑ Caspase-3 ↓
[161]	BDNF-AS ↑	-	Bax ↑ Bcl-2 ↓ Caspase-3 ↑	miR-125b-5p ↑ BDNF-AS ↓	Bax ↓ Bcl-2 ↑ Caspase-3 ↓
[162]	BDNF ↓		Bax ↑ Bcl-2 ↓	miR-22	BDNF ↑ Bax ↓ Bcl-2 ↑
[163]	BDNF ↓	-	Bax ↑ Bcl-2 ↓ Caspase-3 ↑	Mesenchymal stem cell application	BDNF ↑ Bax ↓ Bcl-2 ↑ Caspase-3 ↓
[164]	BDNF ↓	-	Bax ↑ Bcl-2 ↓ Caspase-3 ↑	Neural stem cells	BDNF ↑ Bax ↓ Bcl-2 ↑ Caspase-3 ↓
[165]	BDNF ↓	-	Positive apoptotic neuronal cells ↑	BDNF-modified hUC-MSCs	BDNF ↑ Positive apoptotic neuronal cells ↓
[166]	BDNF ↓	TrkB ↓	Bax ↑ Bcl-2 ↓ Caspase-3 ↑ Caspase-9 ↑	*Cynomorium songaricum* (antioxidant, anti-inflammatory)	BDNF ↑ TrkB ↑ Bax ↓ Bcl-2 ↑ Caspase-3 ↓ Caspase-9 ↓
[167]	BDNF ↓	-	Bax ↑ Bcl-2 ↓ Caspase-3 ↑ Caspase-9 ↑	Carbenoxolone (anti-inflammatory)	BDNF/TrkB/CREB pathway ↑ Bax ↓ Bcl-2 ↑ Caspase-3 ↓ Caspase-9 ↓
[168]	BDNF ↓	-	DNA fragmentation ↑	*Lactuca capensis* (anti-inflammatory)	BDNF ↑ DNA fragmentation ↓
[169]	BDNF ↓	TrkB ↓	DNA fragmentation ↑	*Astragaloside* Exercise rehabilitation	BDNF ↑ TrkB ↑ DNA fragmentation ↓
[170]	BDNF ↓	TrkB ↓	Bax ↑ Bcl-2 ↓ Pro-caspase-3 ↓ Active caspase-3 ↑	Icariside II (antioxidant, anti-inflammatory)	BDNF/TrkB/CREB pathway ↑ Bax ↓ Bcl-2 ↑ Pro-caspase-3 ↑ Active caspase-3 ↓
[171]	BDNF ↓	TrkB ↓	Number of neuronal Nissl bodies ↓	EPA-enriched ethanolamine plasmalogen EPA-enriched phosphatidylethanolamine	BDNF/TrkB/CREB pathway ↑ Number of neuronal Nissl bodies ↑
[172]	BDNF ↓	TrkB ↓	Bax ↑ Bcl-2 ↓	Naringin (antioxidant, anti-apoptotic, anti-inflammatory)	BDNF ↑ TrkB ↑ Bax ↓ Bcl-2 ↑
[173]	BDNF ↓	TrkB ↓	Neuronal apoptosis in CA1 ↑	Tan II (antioxidant, anti-apoptotic, anti-inflammatory)	BDNF/TrkB/CREB pathway ↑
[174]	BDNF ↓	TrkB ↑	Bax ↑ Bcl-2 ↓ Caspase-3 ↑	Syringic acid (antioxidant, anti-apoptotic, anti-inflammatory)	BDNF/TrkB/CREB pathway ↑ Bax ↓ Bcl-2 ↑ Caspase-3 ↓
[175]	BDNF ↓	-	Caspase-3 ↑	Diminazene aceturate	BDNF ↑ Caspase-3 ↓
[176]	BDNF ↓	-	Bax ↑ Bcl-2 ↓ Caspase-3 ↑	L-theanine (antioxidant, anti-inflammatory)	BDNF ↑ Bax ↓ Bcl-2 ↑ Caspase-3 ↓

**Table 3:** The implications of the BDNF/proBDNF relationship in apoptotic-related AD-like disorders in mouse experimental models.

	BDNF/proBDNF	Neurotrophin Receptor	Apoptotic Mechanism	Treatment	Effect
[177]	BDNF ↓	TrkB ↓	Neuronal nuclear protein ↓ ↑ Apoptosis	-	-
[178]	BDNF ↓	-	Caspase-3 ↑	Curcumin (anti-inflammatory, neuroprotective)	BDNF ↑ Caspase-3 ↓
[179]	BDNF ↓	-	Number of apoptotic cells ↑ Amyloid accumulation ↑	Polysaccharide fraction of *Phellinus ribis* (neurotrophic, neuroprotective)	BDNF ↑ Number of apoptotic cells ↓ Amyloid accumulation ↓
[180]	BDNF ↓	TrkB ↓	Bcl-2/Bax ratio ↓	DL0410 (AChE inhibitor, antioxidant, anti-inflammatory)	BDNF ↑ TrkB ↑ Bcl-2/Bax ratio ↑
[181]	BDNF ↓	-	Neuronal cell loss and injury ↑ Bax/Bcl-2 ratio ↑	3,6′-Disinapoyl sucrose (antidepressant, antioxidant)	BDNF ↑ Neuronal cell loss and injury ↓ Bax/Bcl-2 ratio ↓
[182]	BDNF ↓	-	Apoptosis signal-regulating kinase-1 ↑ c-Jun N-terminal kinase mRNA ↑	Qifu Yin (antioxidant, anti-apoptotic)	BDNF ↑ Apoptosis signal-regulating kinase-1 ↓ c-Jun N-terminal kinase mRNA ↓
[183]	BDNF ↓	-	Caspase-3 ↑ Bax ↑ Phosphorylated p53 ↑	(Gene regulating) conversion of PUFA-6 to PUFA-3	BDNF ↑ Caspase-3 ↓ Bax ↓ Phosphorylated p53 ↓
[184]	BDNF ↓	TrkB ↓	Bax ↑ Bcl-2 ↓ Caspase-3 ↑	Cycloastragenol (antioxidant, anti-apoptotic, anti-inflammatory)	BDNF ↑ TrkB ↑ Bax ↓ Bcl-2 ↑ Caspase-3 ↓
[185]	BDNF ↓	-	Caspase-3 ↑	Vanillin (antioxidant)	BDNF ↑ Caspase-3 ↓
[186]	BDNF ↓	-	Caspase-3 ↑ Caspase-7 ↑ Caspase-9 ↑ Adam-10 ↑	Resveratrol (antioxidant) Exercise training	BDNF ↑ Caspase-3 ↓ Caspase-7 ↓ Caspase-9 ↓ Adam-10 ↓
[187]	proBDNF ↑ BDNF ↓	TrkB ↓	Neuronal cell loss and injury ↑ JNK/c-Jun ↑	PAI-039	proBDNF ↓ BDNF ↑ TrkB ↑ Neuronal cell loss and injury ↓ JNK/c-Jun ↓
[188]	proBDNF ↑ BDNF ↓	TrkB ↓	tPA/mBDNF/TrkB signaling pathway ↑ proBDNF/BDNF ↑	-	-
[189]	BDNF ↓	-	Bax ↑ Bcl-2 ↓ Pro-caspase-3 ↑ Caspase-3 ↑	Melatonin (antioxidant, anti-apoptotic, anti-inflammatory)	Akt/ERK/CREB signaling Bax ↓ Bcl-2 ↑ Pro-caspase-3 ↓ Caspase-3 ↓
[190]	BDNF ↓	-	Bax ↑ Bcl-2 ↓ Caspase-3 ↑ Hippocampal BDNF-immunoreactive neurons ↓	*Ginkgo biloba* L. leaf extract *Hericium erinaceus* (Bull.) Pers. fruit extract (antioxidant, anti-inflammatory)	BDNF ↑ Bax ↓ Bcl-2 ↑ Caspase-3 ↓ Hippocampal BDNF-immunoreactive neurons ↑
[191]	BDNF ↓	-	Adam-10 ↓ Presenilin-1 ↑ β-secretase-1 ↑	Propane-2-sulfonic acid octadec-9-enyl-amide (antioxidant, anti-inflammatory, anti-apoptotic)	BDNF ↑ Presenilin-1 ↓ β-secretase-1 ↓ Adam-10 ↑
[192]	BDNF ↓		Fas-L ↑	Human umbilical cord blood-derived stem cells	BDNF ↑ Fas-L ↓ CREB ↑
[193]	proBDNF ↑ BDNF ↓	pTrkB ↑ TrkB ↓ p75 ↑	Hippocampal neuronal cell loss ↑	Electroacupuncture	proBDNF ↓ BDNF ↑ TrkB ↑ pTrkB ↓ p75 ↓ Hippocampal neuronal cell loss ↓

**Table 6:** The implications of the BDNF/proBDNF relationship in apoptotic-related PD-like disorders in in vitro PD models.

	BDNF/proBDNF	Neurotrophin Receptor	Apoptotic Mechanism	Treatment	Effect
[226] (rat mesencephalic dopamine neurons and SH-SY5Y cell line)	BDNF ↓	TrkB ↓	Cell viability↓ Apoptotic rate ↑	THSG (antioxidant, anti-aging, anti-inflammatory)	BDNF ↑ Apoptotic rate ↓ Bcl-2 ↑ Cleaved caspase-3 ↓
[227] (human neuroblastoma SK-N-SH and CHP 212 cells)	BDNF ↓	-	Bax ↑ Bcl-2 ↓ Cleaved caspase-3 ↑	Pramipexole (PPX) (selective dopamine D2 receptor agonist)	BDNF↑ Bax ↓ Bcl-2 ↑ Cleaved caspase-3 ↓
[228] (rat ventral mesencephalon and dopaminergic cell line MN9D)	BDNF ↓	-	Apoptotic rate ↑	Norepinephrine	BDNF ↑ Tyrosine hydroxylase ↑ pERK1/2 ↑
[204] (human monocytic THP-1 cells, murine BV-2 microglia cell line, murine NSC-34 neuronal cells, and cDNA from human primary microglia and astrocytes)	-	-	Caspase-3/7 ↑	GLP-1 (glucagon-like peptide-1), GIP (glucose-dependent insulinotropic polypeptide)	BDNF ↑ Caspase-3/7 ↓
[229] (SH-SY5Y cell line)	BDNF ↓	-	Mitochondria damage ↑ Cell viability ↓	EPA (eicosapentaenoic acid)	BDNF ↑ Mitochondria damage ↓ Cell viability ↑
[230] (HT-29 (human colon adenocarcinoma) and SH-SY5Y)	BDNF ↓	-	Bax/Bcl-2 ratio ↑ Caspase-3 ↑	*Lactobacillus plantarum 200655*	BDNF ↑ Bax/Bcl-2 ratio ↓ Caspase-3 ↓ Apoptotic rate ↓
[231] (SH-SY5Y cells and murine macrophage cell line RAW 264.7)	BDNF ↓	-	Bcl-2 ↓ Cytochrome c ↑	Hydroxy-4′-methoxychalcone (AN07), a synthetic chalcone derivative	BDNF ↑ Bcl-2 ↑ Cytochrome c ↓

**Table 7:** The implications of the BDNF/proBDNF relationship in apoptotic-related HD-like disorders in animal models.

	BDNF/proBDNF	Neurotrophin Receptor	Apoptotic Mechanism	Treatment	Effect
[232] (pro-BDNF knockout mouse model)	BDNF ↓	TrkB n.c. p75 n.c. sortilin n.c.	Caspase-3 ↑ Bcl-2 ↓ Cell density ↓ Nuclear pyknosis ↑	-	-
[233] (Q175 knock-in mouse model)	-	TrkB n.c. p75 n.c.	Bcl-XL ↓ XIAP ↓ Striatal volume ↓	-	-
[234] (R6/2 mutant mice)	BDNF ↓	-	Bax/Bcl-2 ratio ↑ Striatal volume ↓ Number of striatal neurons ↓	INO-1001 (PARP-1 inhibitor)	BDNF ↑ Bax/Bcl-2 ratio ↓ Striatal volume ↑ Number of striatal neurons ↑
[235] (R6/2 mutant mice)	BDNF ↓	-	Number of striatal neurons ↓ Granule cell survival ↓	Exogenous BDNFE7	BDNF ↑ Number of striatal neurons ↑ Granule cell survival n.c.
[236] (R6/1 mutant mice)	BDNF ↓		Caspase-3 ↑	PACAP	BDNF ↑ Caspase-3 ↓
[237] (3-NP mouse and cell line model)	BDNF ↓	pTrkB/TrkB ↓	Caspase-3 ↑ Number of striatal neurons ↓	7,8-DHF (TrkB agonist)	BDNF ↑ pTrkB/TrkB ↑ Caspase-3 ↓ Number of striatal neurons ↑
[238] (3-NP mouse model)	BDNF ↓	-	Bax ↑ Caspase-3 ↑ Bcl-2 ↓ Striatal nuclear pyknosis ↑	Rutin and selen (antioxidant, anti-apoptotic, anti-inflammatory)	BDNF ↑ Bax ↓ Bcl-2 ↑ Caspase-3 ↓ Striatal nuclear pyknosis ↓
[239] (3-NP rat model)	BDNF ↓	-	Bax ↑ p53 ↑ Bcl-2 ↓ Number of striatal neurons ↓	Diapocynin (NADPH oxidase inhibitor)	BDNF ↑ Bax ↓ Bcl-2 ↑ p53 ↓ Number of striatal neurons ↑
[240] (3-NP rat model)	BDNF ↓	pTrkB/TrkB ↓	Caspase-3 ↑ Bcl-2 ↓ Striatal lesion score ↑	Berberine (anti-inflammatory, antioxidant)	BDNF ↑ pTrkB/TrkB ↑ Caspase-3 ↓ Bcl-2 ↑ Striatal lesion score ↓
[241] (3-NP on SH-SY5Y cells)	BDNF ↓	TrkB ↓	Caspase-3 ↑ Apoptosis (flow cytometry) ↑	4-MTBITC (structural analog of sulforaphane)	BDNF TrkB ↑ Caspase-3 ↓ Apoptosis (flow cytometry) ↓

**Table 8:** Therapeutic approaches targeting the BDNF/proBDNF ratio: mechanisms and limitations.

Therapeutic Strategy	Mechanism of Action	Targeted Pathway	Key Limitations
BDNF Gene Therapy [244]	AAV viral vector-mediated overexpression of BDNF	Endogenous BDNF/TrkB signaling	Delivery precision, safety concerns, immune response
TrkB Agonists [249,251]	Mimic BDNF and selectively activate TrkB signaling	PI3K/Akt and MAPK/ERK survival pathways	Poor pharmacokinetics, short half-life, limited BBB permeability
p75NTR Antagonists [252]	Block proBDNF-induced apoptosis	JNK and caspase-3 pro-apoptotic pathway	Interference with normal neurodevelopment and plasticity
Protease Activators [248]	Enhance conversion of proBDNF to BDNF	BDNF maturation and PI3K/Akt survival pathway	Limited specificity, risk of off-target shedding, lack of brain-selective activators
Sortilin Inhibitors [99]	Prevent formation of proBDNF–p75NTR apoptotic complex	ProBDNF binding and apoptotic pathway inhibition	Lack of clinical-grade inhibitors, unknown long-term safety
miRNA Modulation (e.g., miR-125b-5p) [161]	Restore BDNF translation by inhibiting BDNF-suppressing miRNAs	Post-transcriptional regulation and inhibition of Bax/Bcl-2 mitochondrial apoptosis	Delivery challenges, off-target gene effects

## Abbreviations

The following abbreviations are used in this manuscript:

BDNF	Brain-derived neurotrophic factor
PI3K/Akt	Phosphatidylinositol 3-kinase/Protein kinase B
MAPK/ERK	Mitogen-activated protein kinase/Extracellular signal-regulated kinase
JNK	Jun N-terminal kinase
Rho	RAS homolog
GTPase	Small or monomeric guanine nucleotide-binding regulatory proteins
TRAF6	TNF receptor-associated factor 6
AD	Alzheimer’s disease
PD	Parkinson’s disease
HD	Huntington’s disease
NTFs	Neurotrophic factors
CNS	Central nervous system
PNS	Peripheral nervous system
NGF	Nerve growth factor
CDNF/MANF	Cerebral dopamine neurotrophic factor/Mesencephalic astrocyte-derived
NT	Neurotrophin
Trk	Tropomyosin receptor kinase
P75NTR	P75 neurotrophic receptor
PLCγ	Phospholipase C-γ
TNF	Tumor necrosis factor
NFkB	Nuclear factor kappa-light-chain-enhancer of activated B cells
MAG	Myelin-associated glycoprotein
Omg	Oligodendrocyte-myelin glycoprotein
Bcl-2	B-cell lymphoma 2
BAX	BCL2-associated X
PTB	Phosphotyrosine-binding
SH-2	src-homology-2
DAG/IP	Diacylglycerol/Inositol trisphosphate
GRB2/SOS	Growth-factor-receptor-binding protein 2/Son of Sevenless
IRS1	Insulin receptor substrate 1
PDK1	Pyruvate dehydrogenase kinase 1
PIP2	Phosphatidylinositol-4,5-bisphosphate
PIP3	Phosphatidylinositol-3,4,5-trisphosphate
PDK	Phosphoinositide-dependent kinase-1
mTORC2	Mammalian target of rapamycin complex 2
GSK3β	Glycogen synthase kinase 3 beta
FOXO-1	Forkhead box protein O1
CREB	Cyclic AMP-responsive element-binding protein
PCNA	Proliferating cell nuclear antigen
NMDA-R	N-methyl-D-aspartate receptor
RSKs	Ribosomal S6 kinases
MAPKAPK2	MAP kinase-activated protein kinase 2
ATF2	Activating transcription factor 2
PKC	Protein kinase C
SGCs	Satellite glial cells
mRNAs	Non-coding RNAs
CRTC1	CREB-regulated transcription coactivator-1
CBP	CREB-binding protein
DMN	Default mode network
MEF-2	Myocyte enhancer factor 2
PAI-1	Plasminogen activator inhibitor-1
ApoE	Apolipoprotein E
SPNs	Spiny projection neurons
BAI1	Brain-specific angiogenesis inhibitor-1
STAT3/EZH2	Signal transducer and activator of transcription 3/Enhancer of zeste homolog 2
BDNF-AS	lncRNA BDNF antisense
ΔΨm	Mitochondrial membrane potential
MWM	Morris water maze
MPP	1-Methyl-4-phenyl-pyridinium
MPTP	1-Methyl-4-phenyl-1,2,3,6-tetrahydropyridine
MMPs	Matrix metalloproteinases
EPA-pPE	EPA-enriched ethanolamine plasmalogen
EPA-PEEPA	Enriched phosphatidylethanolamine
AchE	Acetylcholinesterase
DISS-3,6′	Disinapoyl sucrose P
PUFA	Polyunsaturated fatty acid
APP/PS1	Double-transgenic mice expressing a chimeric mouse/human amyloid precursor protein (Mo/HuAPP695swe) and a mutant human presenilin 1 (PS1-dE9)
α-GPC	Choline alfoscerate
PPARγ	Proliferator-activated receptor
FXR	Farnesoid X receptor
GLP-1	Glucagon-like peptide-1
GIP	Glucose-dependent insulinotropic polypeptide
6-OHDA	6-Hydroxydopamine hydrobromide
Nrf2	Nuclear factor erythroid 2-related factor 2
THSG	2,3,5,4′-Tetrahydroxystilbene-2-O-β-D-glucoside
FGF2	Fibroblast growth factor 2
PPX	Pramipexole
EPA	Eicosapentaenoic acid
DHA	Docosahexaenoic acid
GDN	Lacrimal glial cell line-derived neurotrophic factor
LPSs	Lipopolysaccharides
XIAP	X-linked inhibitor of apoptosis protein
PARP1	Poly(ADP-ribose) polymerase
PACAP	Pituitary adenylate cyclase-activating polypeptide
PAC1	Pituitary AC-activating polypeptide (PACAP) receptor (ADCAYP1R1)
7,8-DHF	7,8-Dihydroxyflavone
3-NP	3-Nitropropionic acid
4-MTBITC	4-(Methylthio)butyl isothiocyanate
